# Socioeconomic and gendered inequities in travel behaviour in Africa: Mixed-method systematic review and meta-ethnography

**DOI:** 10.1016/j.socscimed.2021.114545

**Published:** 2022-01

**Authors:** Louise Foley, Anna Brugulat-Panés, James Woodcock, Ishtar Govia, Ian Hambleton, Eleanor Turner-Moss, Ebele R.I. Mogo, Alice Charity Awinja, Philip M. Dambisya, Sostina Spiwe Matina, Lisa Micklesfield, Safura Abdool Karim, Lisa Jayne Ware, Marshall Tulloch-Reid, Felix Assah, Caitlin Pley, Nadia Bennett, Georgina Pujol-Busquets, Kufre Okop, Tanmay Anand, Camille M. Mba, Haowen Kwan, Gudani Mukoma, Megha Anil, Lambed Tatah, Lee Randall

**Affiliations:** a MRC Epidemiology Unit, University of Cambridge, Cambridge, United Kingdom; b Caribbean Institute for Health Research, The University of the West Indies, Kingston, Jamaica; c George Alleyne Chronic Disease Research Centre, Caribbean Institute of Health Research, The University of the West Indies, Bridgetown, Barbados; d Adaptive Management Research Consultancy, Kisumu, Kenya; e Health Policy and Systems Division, School of Public Health and Family Medicine, University of Cape Town, Cape Town, South Africa; f SAMRC-Wits Developmental Pathways for Health Research Unit, Faculty of Health Sciences, University of the Witwatersrand, Johannesburg, Gauteng, South Africa; g DSI-NRF Centre of Excellence in Human Development, University of the Witwatersrand, Johannesburg, Gauteng, South Africa; h SAMRC Centre for Health Economics and Decision Science – PRICELESS SA, University of the Witwatersrand, Johannesburg, Gauteng, South Africa; i Health of Populations in Transition (HoPiT) Research Group, Faculty of Medicine and Biomedical Sciences, The University of Yaoundé I, Yaoundé, Cameroon; j School of Clinical Medicine, University of Cambridge, Cambridge, United Kingdom; k Division of Exercise Science and Sports Medicine, Department of Human Biology, Faculty of Health Sciences, University of Cape Town, Cape Town, South Africa; l Faculty of Health Sciences, Universitat Oberta de Catalunya (Open University of Catalonia, UOC), Barcelona, Spain; m Research Centre for Health Through Physical Activity, Lifestyle and Sport (HPALS), ESSM, FIMS International Collaborating Centre of Sports Medicine, Department of Human Biology, Faculty of Health Sciences, University of Cape Town, Cape Town, South Africa; n Centre for Social Science Research (CSSR), Faculty of Humanities, University of Cape Town, Cape Town, South Africa

**Keywords:** Systematic review, Meta-ethnography, Travel, Equity, Socioeconomic status, Gender, Africa

## Abstract

Travel has individual, societal and planetary health implications. We explored socioeconomic and gendered differences in travel behaviour in Africa, to develop an understanding of travel-related inequity. We conducted a mixed-methods systematic review (PROSPERO CRD42019124802). In 2019, we searched MEDLINE, TRID, SCOPUS, Web of Science, LILACS, SciELO, Global Health, Africa Index Medicus, CINAHL and MediCarib for studies examining travel behaviour by socioeconomic status and gender in Africa. We appraised study quality using Critical Appraisal Skills Programme checklists. We synthesised qualitative data using meta-ethnography, followed by a narrative synthesis of quantitative data, and integrated qualitative and quantitative strands using pattern matching principles. We retrieved 103 studies (20 qualitative, 24 mixed-methods, 59 quantitative). From the meta-ethnography, we observed that travel is: intertwined with social mobility; necessary to access resources; associated with cost and safety barriers; typified by long distances and slow modes; and dictated by gendered social expectations. We also observed that: motorised transport is needed in cities; walking is an unsafe, ‘captive’ mode; and urban and transport planning are uncoordinated. From these observations, we derived hypothesised patterns that were tested using the quantitative data, and found support for these overall. In lower socioeconomic individuals, travel inequity entailed reliance on walking and paratransit (informal public transport), being unable to afford travel, travelling less overall, and travelling long distances in hazardous conditions. In women and girls, travel inequity entailed reliance on walking and lack of access to private vehicles, risk of personal violence, societally-imposed travel constraints, and household duties shaping travel. Limitations included lack of analytical rigour in qualitative studies and a preponderance of cross-sectional quantitative studies (offering a static view of an evolving process). Overall, we found that travel inequity in Africa perpetuates socioeconomic and gendered disadvantage. Proposed solutions focus on improving the safety, efficiency and affordability of public transport and walking.

## Highlights

• Utilised meta-ethnography and pattern-matching principles.
• Revealed travel patterns differed by socioeconomic status and gender.
• Travel inequity compounded disadvantage.
• Females and poor people more likely to rely on walking.
• Cost, safety and cultural factors were barriers to travel.

## 1 Introduction

Mobility refers to the movement of people, objects and information (Sheller, 2014). It incorporates the social mobility of individuals and groups, as well as their physical mobility which we refer to as travel behaviour. Travel behaviour considers the ways in which people move through time and space, their reasons for doing so, and the individual and wider circumstances in which physical movement takes place (Van Acker et al., 2010).

Travel behaviour has individual, societal and planetary health implications. In 2015, the United Nations Sustainable Development Goals (SDGs) set a plan of action for people, planet and prosperity. SDG target 11.2 aims to provide access to “safe, affordable, accessible and sustainable transport systems for all” (United Nations General As, 2015). Though target 11.2 focuses mainly on public transport systems, walking and cycling for transport are also core features of sustainable transport systems. This is due to the health and climate co-benefits achieved through increased population physical activity and reductions in greenhouse gas emissions concurrent with reductions in private motorised transport (Shaw et al., 2014; Sá et al., 2017; Brand et al., 2021).

Travel needs, conditions and behaviours are not uniform within societies. In particular, the travel patterns of lower socioeconomic groups, and women and girls, typically differ from their counterparts in ways that reflect, generate and compound disadvantage worldwide (Di Ciommo and Shiftan, 2017). Recognition of these differences is essential for planning and delivering economically, environmentally and socially sustainable transport systems (Lucas et al., 2016). In low- and middle-income countries (LMICs) including countries in Africa, the evidence base is less developed than in high-income countries, but similarly indicates travel behaviour differs in these groups (Porter, 2008; Sietchiping et al., 2012). Africa faces challenges related to high levels of absolute (43%) (Beegle et al., 2016) and relative (49% in sub-Saharan Africa) (Jolliffe and Prydz, 2021) poverty coupled with rapid and mostly unplanned urbanisation (Cobbinah et al., 2015). This has facilitated the development of a large informal economy (International Labour Organization, 2018) which includes transport. Motorisation rates in Africa are low but increasing rapidly (World Bank, 2019), with the majority of growth in used (rather than new) vehicles (United Nations Environment Programme, 2020). Approximately 80% of urban residents do not have access to private vehicles, leading to reliance on non-motorised modes such as walking, as well as formal and informal public transport (United Nations Habitat, 2010). An understanding of how travel behaviour is embedded in these contextual conditions will facilitate integrated urban and transport planning, and ultimately help to build sustainable and equitable transport systems in Africa.

In this review, we draw together the related ideas of transport poverty (Lucas et al., 2016), transport disadvantage (Kamruzzaman et al., 2016), transport inequality (Banister, 2018), transport inequity (Di Ciommo and Shiftan, 2017) and transport-related exclusion (Benevenuto and Caulfield, 2019) under the umbrella term *travel inequity* to describe unfair variations in travel behaviour between groups. These variations relate to distances required to be traversed and travel time, inability to meet the cost of transport, lack of access to (motorised) transport, lack of safe and efficient non-motorised transport options, difficulty reaching key locations to access opportunities or services, exclusion from transport modes or public places, and exposure to harmful aspects of the transport system such as crime, road traffic danger, pollution and onerous levels of load-carrying. We aim to explore the complex, intertwined and embedded conditions in which travel inequity arises and thus identify context-sensitive avenues for intervention applicable to local and regional policy and practice. The research questions are:

1. What factors shape travel inequity in disadvantaged populations (low socioeconomic status groups, women and girls) in Africa, and how is travel inequity experienced?
2. What are the typical travel patterns (amount and mode choice) associated with travel inequity in these populations?
3. How might travel inequity be addressed?

## 2 Methods

### 2.1 Systematic review design

This mixed-method systematic review and meta-ethnography was conducted in accordance with SAGER (Heidari et al., 2016), PRISMA (Moher et al., 2009), eMERGe (France et al., 2019) and SWiM (Campbell et al., 2020) guidelines (S1 Appendix), and registered with PROSPERO (CRD42019124802). We chose meta-ethnography as this aligned with our broader ambition towards ‘holistic sense making’ (Ogilvie et al., 2020), embracing the unique contribution of quantitative and qualitative perspectives.

This analysis is drawn from a parent review which aimed to examine factors associated with travel behaviour in Africa and the Caribbean. Here we focus on differences in travel behaviour by socioeconomic status and gender in Africa.

### 2.2 Systematic review theoretical framework

The mobilities paradigm (Sheller, 2014) informed our identification of key constructs and the interpretation of findings. This theory is drawn from sociology and considers the practices, meanings and power relations associated with mobility. As described, travel behaviour is a type of physical mobility, but this paradigm also considers the relationship between physical and social mobility.

### 2.3 Study eligibility criteria

We included literature on regular travel behaviour, as this is likely to have a significant impact on health over the long term. We focussed on post-2008 literature to maximise relevance to the current policy context. Studies exploring only school-related travel were excluded as this was the topic of a recent systematic review (Larouche et al., 2014).

Socioeconomic status was operationalised in accordance with recent definitions (Howe et al., 2012) and could incorporate one or any combination of: household assets or characteristics (e.g. vehicle ownership, electricity or water supply), education, income, occupation or employment status. Gender was operationalised as incorporating both biological sex (as typically reported by quantitative studies) as well as psychosocial and cultural factors influencing gender identity, which were more commonly explored in the qualitative literature. We were not able to disaggregate sex- and gender-specific relationships, as this distinction was not binary (e.g. quantitative studies typically assessed sex, but identified relationships with travel behaviour that were likely gendered). The eligibility criteria are detailed in Table 1.

**Table 1:** Systematic review inclusion and exclusion criteria.

Domain	Inclusion criteria	Exclusion criteria
Study design	Studies must contain empirical data (primary or secondary) and present an analysis of these data. All study designs (quantitative and qualitative) are eligible	Literature reviews, narrative overviews, commentaries, opinion pieces, or any format not providing sufficient information to allow for data extraction
Participants	The general population living in African and Caribbean countries. No age or sex/gender restrictions For this analysis we focussed on Africa only	Studies focussed on specific or unique population segments in which travel is likely to be atypical: people with specific health conditions; professional travellers (e.g. bus drivers, professional cyclists); tourists, refugees, asylum seekers or migrants, victims of trafficking Studies investigating non-human travel such as food or freight
Exposures	Both correlates (where causality is uncertain), as well as purported causal influences on travel behaviour For this analysis we focussed on socioeconomic status and gender only. Socioeconomic status incorporated any of household assets or characteristics, education, income, occupation or employment status. Gender incorporated male or female, or social constructions of biological sex	
Comparators	All eligible, if used	
Outcomes	Routine or regular travel behaviour, including: time spent in all travel or particular travel modes; number of trips; choice or use of particular travel modes or combinations of modes; mode share	Studies focussed on single travel purposes: school-related travel; travel to administer or receive healthcare Studies focussed on hypothetical (rather than actual) use of transport modes Studies without a primary focus on travel *per se*: road traffic accidents, injuries or road safety as the main outcome
Timing	January 1, 2008–January 31, 2019	
Setting	Africa and the Caribbean For this analysis we focussed on Africa only (see S2 Appendix)	Studies set in contexts in which travel is likely to be atypical: war, political crises or natural disasters
Language	All languages considered	

### 2.4 Information sources and search methods for study identification

We searched MEDLINE, Transport Research International Documentation (TRID), SCOPUS, Web of Science, LILACS, SciELO, Global Health, Africa Index Medicus, CINAHL and MediCarib. These databases were selected to cover relevant academic disciplines (i.e. health, transport and the social sciences) and geographical areas. Following a pilot in December 2018, final database searches were conducted in February 2019 in consultation with a medical librarian (S3 Appendix).

### 2.5 Study selection

Using Covidence software (Covidence, 2021), citations were imported and duplicates removed. Titles and abstracts, and full-texts, were 100% double screened against the eligibility criteria by junior team members who had received training. Discrepancies were resolved by senior team members (S4 Appendix). Although excluded, literature reviews were mined for additional citations. Forward (citation screening using Scopus and Web of Science) and backward (reference list) screening of eligible studies was undertaken. These additional citations were single screened by a senior member. In November 2020, 27 topic experts identified from the database search were invited to provide additional citations, of whom eight responded. These additional citations were single screened by a senior member. This resulted in the final set of eligible studies. For the current analysis, we selected studies from the final set that examined differences in travel behaviour by socioeconomic status and gender.

### 2.6 Study data extraction and data items

A data extraction template (S5 Appendix) was created, piloted and refined by senior members. Using Covidence, data extraction took place in two stages. Initially, eligible studies were extracted by junior members who had received training. Following this, senior members double extracted selected fields for a randomly selected 20% of eligible studies. Due to the heterogeneity of the literature identified, unacceptable levels of disagreement (>50%) were encountered. Consequently, data extraction was reproduced for 100% of the eligible studies by a smaller group of senior members. Discrepancies were resolved by discussion within this group.

For the qualitative data only (i.e. from qualitative and mixed-methods studies), LF, AB and LR undertook close reading of the studies. For each study, this was followed by extraction of the geographic setting, theoretical underpinning, and second order constructs (study author interpretations of participant data) (Toye et al., 2014) into a spreadsheet. Data were extracted from across the full primary study (i.e. not just results or discussion sections). Initially, two qualitative studies were independently extracted by all three group members to test consistency. Procedural issues were resolved through discussion and the remaining studies were extracted by one of LF, AB or LR. Separately, each researcher maintained reflexive notes on different countries’ history, geography and transport systems. These were used to maintain contextual sensitivity but did not form part of the formal data extraction.

### 2.7 Study quality

Critical Appraisal Skills Programme (CASP) checklists (Critical Appraisal Skills Programme, 2021) were used to appraise the trustworthiness, relevance and results of eligible studies. The checklists assess overall methodological rigour as well as (for quantitative designs) risk of bias. For quantitative studies (and the quantitative element of mixed-methods studies), an adapted CASP cohort study checklist was used (S6 Appendix), which included items on selection and measurement biases, confounding and generalisability. For qualitative studies (and the qualitative element of mixed-methods studies), the CASP qualitative checklist was used (S7 Appendix), which included items on the appropriateness of the design, methodology, recruitment and analysis, as well as how ethical issues and the relationship between participant and researcher were considered. Each study was appraised by one senior member. We did not use quality as a basis on which to exclude studies, nor did we apply thresholds to define higher or lower quality. Rather, we used this to identify recurring quality issues across the literature.

In addition, we rated the thickness of qualitative data (Ponterotto, 2006) in terms of interpretation in context; capturing thoughts and emotions; verisimilitude (making the setting ‘come alive’); assigning motivations and intentions; and explaining the meaningfulness of findings (S8 Appendix). Again, we did not apply thresholds to these ratings, but noted that thicker studies tended to assume greater prominence in the analysis as they contributed a greater number of second order constructs.

### 2.8 Synthesis of results

By synthesis, we refer to the process of synthesising study findings within either the qualitative or quantitative strands. For the qualitative data (i.e. from qualitative and mixed-methods studies), second order constructs were used as the basic unit of synthesis. LF, AB and LR began by comparing second order constructs across studies. From there, commonalities emerged, which were iteratively sorted into ‘conceptual clusters’. We began to orient the clusters more specifically against the research questions and to identify, contrast and compare geographical and theoretical elements.

We developed the conceptual clusters into formal third order constructs (reviewer interpretations) (Toye et al., 2014). Given the large number of studies, our reflexive notes were used to remain grounded in the data. We noted both confirming and disconfirming evidence for each third order construct, thus utilising reciprocal and refutational translation (Toye et al., 2014). We gave each third order construct a working title and arranged them relative to each other. Then, we presented a visual map of third order constructs to the wider research team for critique. The researchers spanned multiple academic disciplines (including health, transport and the social sciences) and countries (including six African countries). The group commented on overall coherence and suggested where constructs could be renamed, reordered, split or merged. Based on their contextual and topic knowledge, some researchers commented on surprising or missing findings; in these cases LF, AB and LR went back to the second order constructs to check whether misinterpretations or omissions had been made. Following this, we produced a final visual map of third order constructs.

For the quantitative data, we undertook a narrative synthesis due to marked heterogeneity in study methods and outcomes. We used individual study findings as the basic unit of synthesis, which we considered analogous to the qualitative second order constructs. We described the overall direction of effect for each finding due to methodological heterogeneity. Our synthesis was therefore limited to vote counting based on direction of effect (McKenzie et al., 2019). In terms of study design, quality and risk of bias, our quantitative data were homogenous (the vast majority employing cross-sectional designs and simple descriptive analyses). Therefore, we did not prioritise any particular studies. We analysed sub-groups of interest (i.e. socioeconomic status and gender) separately. Our appraisal of the certainty of evidence was bespoke, as it was not possible to apply existing frameworks such as GRADE (Schünemann et al., 2021). Instead, we used vote counting combined with elements of Bradford Hill's principles of causation (Hill, 1965) to guide us towards findings that were more certain. In particular, we considered the Bradford Hill domains of consistency (similar findings across multiple studies and countries), plausibility (plausible mechanism of cause and effect informed by qualitative synthesis), coherence (similar findings across different disciplines and methods) and analogy (similarities between related findings). From these, we described the overall certainty of findings from least to most as: none, limited, some, clear, strong or compelling, and the direction of effect as either mixed or consistent.

### 2.9 Analysis

By analysis, we refer to the process of integrating the qualitative and quantitative strands. We conducted an exploratory sequential analysis (Fetters et al., 2013). We prioritised the qualitative data and synthesised this first, which informed the subsequent quantitative synthesis.

We used the principles of pattern matching (Trochim, 1989). Theory can be used to generate patterns of predictions. Pattern matching involves the specification of a hypothesised pattern, the acquisition of an observed pattern using empirical data, and an attempt to match the two. This moves beyond single hypothesis testing because the complexity of the pattern is important. If corroborated by the observed pattern, the more complex hypothesised pattern provides a stronger basis for causal inference because it becomes less likely that alternative theories could explain that pattern. For this analysis, we used insights from the synthesis of the qualitative data to generate hypothesised patterns of predictions. We then turned to the quantitative synthesis to see whether these patterns could be observed. In doing so, we did not expect to find uniform behavioural regularities, which would not be appropriate for the culturally, spatially and demographically diverse countries and settings represented in this review. Rather, we used pattern matching to specify a set of outcomes that we predicted would be important for, or common to, the experience of travel inequity in varying degrees across different settings. We remained open to mixed findings (i.e. positive, neutral, negative or a combination of these) across the different hypotheses.

Pattern matching was used to generate our overall line of argument, drawing together all eligible studies (qualitative, mixed-methods and quantitative). This was developed initially by LF, AB and LR. We refined it further with the wider team of researchers and other stakeholders.

### 2.10 Patient and public involvement

The research team brought diverse perspectives, which were broadened through discussions with the wider collaboration (https://www.gdarnet.org/) (Oni et al., 2020). Stakeholders from the Institute for Transportation and Development Policy, World Health Organization and United Nations Human Settlements Programme (UN-HABITAT) were consulted in February 2021 to reflect on emerging findings and help frame contextually realistic and relevant solutions to travel inequity.

## 3 Results

### 3.1 Included studies

**Fig. 1:**
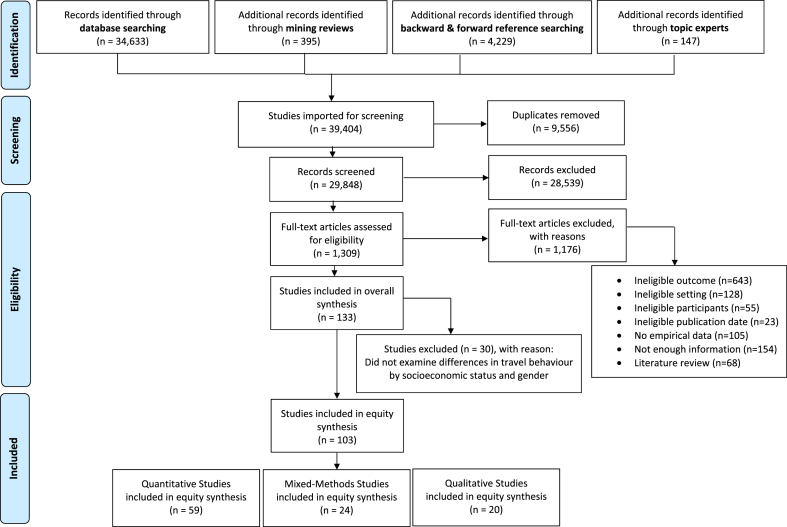
PRISMA flow diagram.

From 39,404 citations, 133 studies met eligibility criteria for the parent review (Fig. 1). For the current analysis, we retrieved a subset of 103 studies: 20 qualitative (Alando and Scheiner, 2016; Archambault, 2012; Benwell, 2009; El-Dorghamy and Mosa, 2016; Esson et al., 2016; Gough, 2008; Gwaka, 2018; Kamuhanda and Schmidt, 2009; Langevang and Gough, 2009; Lesteven and Boutueil, 2018; Lucas, 2011; Poku-Boansi and Cobbinah, 2018; Porter, 2011; Porter et al., 2010a, 2010b; Raynor, 1923; Turner and Adzigbey, 2012; van Blerk, 2013; Yankson et al., 2017; Zolnikov, 2016), 24 mixed-methods (Abane, 2011; Agyemang, 2015; Amoako-Sakyi and Owusu, 2011; Andreasen and Møller-Jensen, 2017; Bogale, 2012; Delatte et al., 2018; Diaz Olvera et al., 2010, 2013; Evans et al., 2018; Irlam and Zuidgeest, 2018; Janusz et al., 2019; Kola et al., 2012; Kumar, 2011; Mbara, 2016; Nkurunziza et al., 2010; Ojo et al., 2014; Oviedo et al., 2017; Integrated Transport Plan, 2010; Porter et al., 2011, 2012, 2013, 2017; Seedhouse et al., 2016; Vermeiren et al., 2015), and 59 quantitative (Lucas, 2011, Lucas et al., 2016, Luke et al., 2014, Machado-León et al., 2017, Malambo et al., 2017, Masaoe et al., 2011, Mbara, 2016, McKenzie et al., 2019, Mfinanga, 2014, Micklesfield et al., 2017, Moher et al., 2009, Muhammed, 2011, Nigatu Haregu et al., 2016, Nkurunziza and van Maarseveen, 2013, Nkurunziza et al., 2010, Odufuwa, 2008, Odufuwa et al., 2012, Ogilvie et al., 2020, Ojo et al., 2014, Olawole, 2015, Olawole, 2017, Olawole and Aloba, 2014, Olawole and Olapoju, 2016, Olojede et al., 2017, Oni et al., 2020, Oviedo et al., 2017, Oyeyemi et al., 2011, Oyeyemi et al., 2016, Oyeyemi et al., 2019, Poku-Boansi and Cobbinah, 2018, Ponterotto, 2006, Porter, 2008, Porter, 2011, Porter et al., 2010a, Porter et al., 2010b, Porter et al., 2011, Porter et al., 2012, Porter et al., 2013, Porter et al., 2017, Priya Uteng and Turner, 2019, Raynor, 1923, Sá et al., 2017, Sabry et al., 2017, Saddier et al., 2016, Salau, 2015, Salon and Aligula, 2012, Salon and Gulyani, 2010, Schalekamp and Behrens, 2010, Schünemann et al., 2021, Schuyler et al., 2017, Seedhouse et al., 2016, Shaw et al., 2014, Sheller, 2014, Sietchiping et al., 2012, Tembe et al., 2018, Toye et al., 2014, Trochim, 1989, Turner and Adzigbey, 2012, United Nations Environment Programme, 2020) (S9 Appendix).

**Fig. 2:**
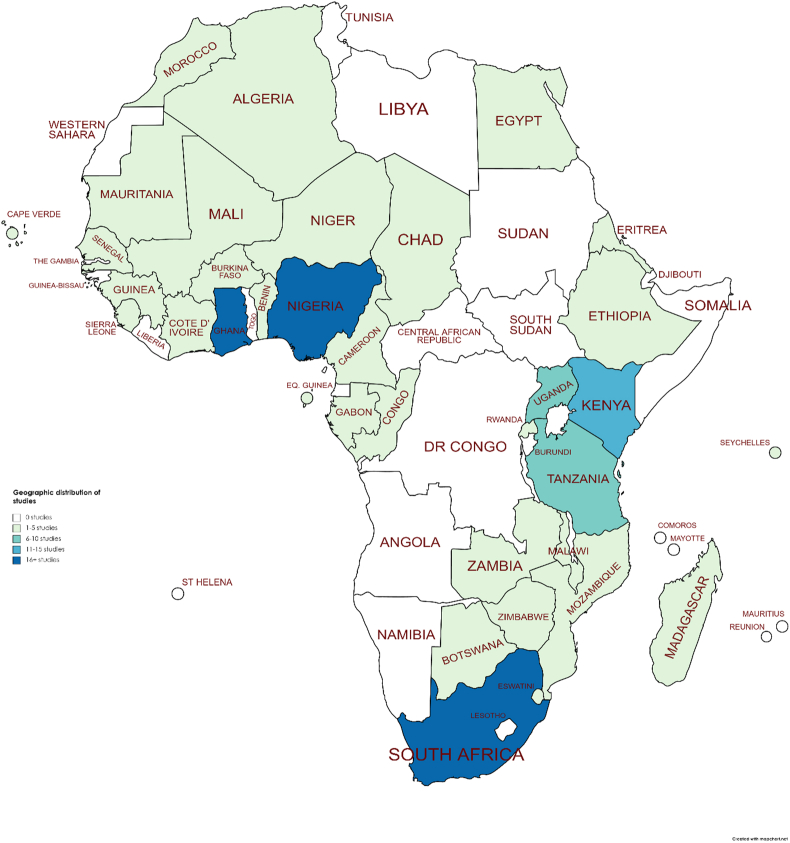
Geographical distribution of included studies. Created using https://mapchart.net/. Licensed under a Creative Commons Attribution-ShareAlike 4.0 International License.

Included qualitative studies were predominantly drawn from anthropology and sociology, and quantitative studies from transport, human geography and health. Studies were retrieved from across the African continent (Fig. 2), but with a marked lack of representation from Central Africa. South Africa (26 studies (Benwell, 2009; Lesteven and Boutueil, 2018; Lucas, 2011; Porter et al., 2010a; Porter et al., 2010b; van Blerk, 2013; Irlam and Zuidgeest, 2018; Mbara, 2016; Porter et al., 2012; Porter et al., 2017; Bartels et al., 2016; Bechstein, 2010; Behrens and Schalekamp, 2010; Chakwizira et al., 2018; Davy et al., 2019; Dugas et al., 2014; Gradidge et al., 2014; Guthold et al., 2011; Kolbe-Alexander et al., 2015; Laverty et al., 2015; Luke et al., 2014; Masaoe et al., 2011; Micklesfield et al., 2017; Venter, 2011; Venter et al., 2011; Walter et al., 2011):), Nigeria (21 studies (Porter, 2011; Kumar, 2011; Oviedo et al., 2017; Seedhouse et al., 2016; Abiola and Ayodeji, 2012; Adetunji, 2012; Alade et al., 2013; Aworemi et al., 2008; Ipingbemi, 2010; Muhammed, 2011; Odufuwa, 2008; Odufuwa et al., 2012; Olawole, 2015; Olawole, 2017; Olawole and Aloba, 2014; Olawole and Olapoju, 2016; Olojede et al., 2017; Oyeyemi et al., 2011; Oyeyemi et al., 2016; Oyeyemi et al., 2019; Salau, 2015):) and Ghana (19 studies (Esson et al., 2016; Langevang and Gough, 2009; Poku-Boansi and Cobbinah, 2018; Porter, 2011; Porter et al., 2010a; Yankson et al., 2017; Abane, 2011; Agyemang, 2015; Amoako-Sakyi and Owusu, 2011; Ojo et al., 2014; Porter et al., 2012; Acheampong, 2016; Acheampong and Siiba, 2018; Agyemang, 2017; Amoh-Gyimah and Aidoo, 2013; Dugas et al., 2014; Laverty et al., 2015; Luke et al., 2014; Saddier et al., 2016):) were most strongly represented. All studies were in English.

Seventy-nine studies (Delatte et al., 2018, Di Ciommo and Shiftan, 2017, Diaz Olvera et al., 2008, Diaz Olvera et al., 2010, Diaz Olvera et al., 2013, Diaz Olvera et al., 2013, Diaz Olvera et al., 2016, El-Dorghamy and Mosa, 2016, El-Sherbiny and Elsary, 2018, Elfiky, 2010, Esson et al., 2016, Evans et al., 2018, Fetters et al., 2013, France et al., 2019, Gough, 2008, Gradidge et al., 2014, Groot and Muthuri, 2017, Guwatudde et al., 2016, Gwaka, 2018, Hill, 1965, Howe et al., 2012, Integrated Transport Plan, 2010, International Labour Orga, 2018, Ipingbemi, 2010, Irlam and Zuidgeest, 2018, Isiagi et al., 2021, Janusz et al., 2019, John et al., 2017, Jolliffe and Prydz, 2021, Joshi et al., 2014, Kamruzzaman et al., 2016, Kamuhanda and Schmidt, 2009, Kola et al., 2012, Kolbe-Alexander et al., 2015, Langevang and Gough, 2009, Loo and Siiba, 2019, Lucas, 2011, Lucas et al., 2016, Luke et al., 2014, Machado-León et al., 2017, Malambo et al., 2017, Masaoe et al., 2011, McKenzie et al., 2019, Mfinanga, 2014, Moher et al., 2009, Muhammed, 2011, Nigatu Haregu et al., 2016, Nkurunziza and van Maarseveen, 2013, Odufuwa, 2008, Odufuwa et al., 2012, Ogilvie et al., 2020, Ojo et al., 2014, Olawole, 2017, Olawole and Olapoju, 2016, Oyeyemi et al., 2011, Oyeyemi et al., 2019, Poku-Boansi and Cobbinah, 2018, Porter, 2011, Porter et al., 2010a, Porter et al., 2010b, Porter et al., 2012, Porter et al., 2013, Porter et al., 2017, Priya Uteng and Turner, 2019, Raynor, 1923, Sá et al., 2017, Saddier et al., 2016, Salau, 2015, Salon and Aligula, 2012, Salon and Gulyani, 2010, Schalekamp and Behrens, 2010, Schünemann et al., 2021, Schuyler et al., 2017, Seedhouse et al., 2016, Shaw et al., 2014, Sheller, 2014, Sietchiping et al., 2012, Toye et al., 2014, Turner and Adzigbey, 2012, United Nations Environment Programme, 2020) were from urban settings, 15 (Amoako-Sakyi and Owusu, 2011; Porter et al., 2011, 2012; Babinard, 2011; Dugas et al., 2014; El-Sherbiny and Elsary, 2018; Groot and Muthuri, 2017; Guthold et al., 2011; Guwatudde et al., 2016; John et al., 2017; Laverty et al., 2015; Luke et al., 2014; Micklesfield et al., 2017; Schuyler et al., 2017; Venter, 2011) from both urban and rural, and eight (Gwaka, 2018; Porter, 2011; Porter et al., 2010b, 2013; Zolnikov, 2016; Seedhouse et al., 2016; Bryceson et al., 2008; Olawole, 2017) from rural settings only (one study (Alemu et al., 2011) did not report urbanity). Eighty-five studies [Delatte et al., 2018, Di Ciommo and Shiftan, 2017, Diaz Olvera et al., 2010, Diaz Olvera et al., 2013, Diaz Olvera et al., 2016, Dugas et al., 2014, El-Dorghamy and Mosa, 2016, El-Sherbiny and Elsary, 2018, Esson et al., 2016, Evans et al., 2018, Factors associa, 1248, Gradidge et al., 2014, Groot and Muthuri, 2017, Guthold et al., 2011, Guwatudde et al., 2016, Gwaka, 2018, Hill, 1965, Howe et al., 2012, Integrated Transport Plan, 2010, International Labour Orga, 2018, Isiagi et al., 2021, Janusz et al., 2019, Joshi et al., 2014, Kamruzzaman et al., 2016, Kamuhanda and Schmidt, 2009, Kola et al., 2012, Langevang and Gough, 2009, Laverty et al., 2015, Lesteven and Boutueil, 2018, Loo and Siiba, 2019, Lucas, 2011, Lucas et al., 2016, Luke et al., 2014, Machado-León et al., 2017, Malambo et al., 2017, Masaoe et al., 2011, Mbara, 2016, McKenzie et al., 2019, Mfinanga, 2014, Micklesfield et al., 2017, Moher et al., 2009, Muhammed, 2011, Nigatu Haregu et al., 2016, Nkurunziza and van Maarseveen, 2013, Odufuwa et al., 2012, Ogilvie et al., 2020, Ojo et al., 2014, Olawole, 2015, Olawole, 2017, Olawole and Aloba, 2014, Olawole and Olapoju, 2016, Olojede et al., 2017, Oni et al., 2020, Oviedo et al., 2017, Oyeyemi et al., 2011, Oyeyemi et al., 2016, Oyeyemi et al., 2019, Poku-Boansi and Cobbinah, 2018, Ponterotto, 2006, Porter, 2008, Porter, 2011, Porter et al., 2010a, Porter et al., 2010b, Porter et al., 2011, Porter et al., 2012, Porter et al., 2013, Porter et al., 2017, Priya Uteng and Turner, 2019, Raynor, 1923, Sá et al., 2017, Sabry et al., 2017, Saddier et al., 2016, Salau, 2015, Salon and Aligula, 2012, Salon and Gulyani, 2010, Schünemann et al., 2021, Schuyler et al., 2017, Shaw et al., 2014, Sheller, 2014, Sietchiping et al., 2012, Tembe et al., 2018, Toye et al., 2014, Trochim, 1989, Turner and Adzigbey, 2012, United Nations Environment Programme, 2020] included adults, 32 included adolescents (Esson et al., 2016; Gough, 2008; Gwaka, 2018; Langevang and Gough, 2009; Lucas, 2011; Porter et al., 2010a; Porter et al., 2010b; van Blerk, 2013; Yankson et al., 2017; Abane, 2011; Agyemang, 2015; Amoako-Sakyi and Owusu, 2011; Bogale, 2012; Delatte et al., 2018; Diaz Olvera et al., 2013; Porter et al., 2012; Porter et al., 2017; Porter et al., 2011; Aworemi et al., 2008; Baouni et al., 2018; Diaz Olvera et al., 2016; El-Sherbiny and Elsary, 2018; John et al., 2017; Machado-León et al., 2017; Micklesfield et al., 2017; Nkurunziza and van Maarseveen, 2013; Oyeyemi et al., 2011; Oyeyemi et al., 2016; Salau, 2015; Schuyler et al., 2017; Tembe et al., 2018; Venter et al., 2011) and 17 included children (Benwell, 2009; El-Dorghamy and Mosa, 2016; Porter, 2011; Porter et al., 2010a, 2010b, 2011, 2012, 2017; van Blerk, 2013; Amoako-Sakyi and Owusu, 2011; Nkurunziza et al., 2010; Bwire, 2011; Elfiky, 2010; Masaoe et al., 2011; Mfinanga, 2014; Salon and Aligula, 2012; Salon and Gulyani, 2010). On examining institutional affiliations, 37 studies (Alando and Scheiner, 2016; Archambault, 2012; Benwell, 2009; El-Dorghamy and Mosa, 2016; Gough, 2008; Langevang and Gough, 2009; Lesteven and Boutueil, 2018; Lucas, 2011; Porter, 2011; Turner and Adzigbey, 2012; van Blerk, 2013; Zolnikov, 2016; Andreasen and Møller-Jensen, 2017; Delatte et al., 2018; Diaz Olvera et al., 2013; Evans et al., 2018; Janusz et al., 2019; Nkurunziza et al., 2010; Oviedo et al., 2017; Integrated Transport Plan, 2010; Porter et al., 2012; Seedhouse et al., 2016; Vermeiren et al., 2015; Acheampong, 2016; Acheampong and Siiba, 2018; Babinard, 2011; Bryceson et al., 2008; Diaz Olvera et al., 2016; Dugas et al., 2014; Guthold et al., 2011; Laverty et al., 2015; Luke et al., 2014; Machado-León et al., 2017; Nkurunziza and van Maarseveen, 2013; Salon and Gulyani, 2010; Schuyler et al., 2017; Tembe et al., 2018) did not have an Africa-based first or last author; 13 studies (Esson et al., 2016; Kamuhanda and Schmidt, 2009; Porter et al., 2010a, 2010b, 2011, 2013, 2017; Diaz Olvera et al., 2010; Baouni et al., 2018; Groot and Muthuri, 2017; Oyeyemi et al., 2011; Saddier et al., 2016; Salon and Aligula, 2012) had an Africa-based researcher as one of these, and 52 studies (Dugas et al., 2014, Evans et al., 2018, France et al., 2019, Groot and Muthuri, 2017, Guwatudde et al., 2016, Gwaka, 2018, Heidari et al., 2016, Howe et al., 2012, Isiagi et al., 2021, John et al., 2017, Joshi et al., 2014, Kamuhanda and Schmidt, 2009, Lucas, 2011, Machado-León et al., 2017, Malambo et al., 2017, Masaoe et al., 2011, McKenzie et al., 2019, Mfinanga, 2014, Muhammed, 2011, Nigatu Haregu et al., 2016, Nkurunziza and van Maarseveen, 2013, Odufuwa, 2008, Odufuwa et al., 2012, Ogilvie et al., 2020, Olawole, 2017, Olawole and Aloba, 2014, Olawole and Olapoju, 2016, Oviedo et al., 2017, Oyeyemi et al., 2011, Oyeyemi et al., 2016, Oyeyemi et al., 2019, Poku-Boansi and Cobbinah, 2018, Porter et al., 2010a, Porter et al., 2010b, Porter et al., 2011, Porter et al., 2012, Porter et al., 2013, Priya Uteng and Turner, 2019, Raynor, 1923, Sá et al., 2017, Sabry et al., 2017, Saddier et al., 2016, Salau, 2015, Salon and Aligula, 2012, Schalekamp and Behrens, 2010, Schünemann et al., 2021, Schuyler et al., 2017, Shaw et al., 2014, Trochim, 1989, Turner and Adzigbey, 2012, United Nations Environment Programme, 2020) had an Africa-based researcher as both (one study (Kumar, 2011) did not report affiliation).

Fifty-five studies did not report funding. For those that did, sources ranged from international agencies (e.g. World Bank, World Health Organization, United Nations), non-African aid or research funds (e.g. United Kingdom Department for International Development, Wellcome Trust, European Union, United States National Institutes of Health) and within-Africa public or research funds (e.g. South African Medical Research Council, South Africa Department of Transport). Five studies reported automobile industry funding (Volvo or Renault).

### 3.2 Quality appraisal

As a whole, studies containing qualitative data clearly stated aims and used data collection methodologies appropriate to the research question. However, details regarding participant recruitment and analytical methods were typically insufficient. Where described, analytical methods appeared to lack rigour and were not often linked to specific qualitative research designs (Creswell and Poth, 2018). Of particular concern was the lack of detail on how ethical issues, and the relationship between participant and researcher, had been considered. In light of the fact that 12 (Alando and Scheiner, 2016; Archambault, 2012; Benwell, 2009; El-Dorghamy and Mosa, 2016; Gough, 2008; Langevang and Gough, 2009; Lesteven and Boutueil, 2018; Lucas, 2011; Porter, 2011; Turner and Adzigbey, 2012; van Blerk, 2013; Zolnikov, 2016) out of 20 qualitative studies, and 11 (Andreasen and Møller-Jensen, 2017; Delatte et al., 2018; Diaz Olvera et al., 2013; Evans et al., 2018; Janusz et al., 2019; Nkurunziza et al., 2010; Oviedo et al., 2017; Integrated Transport Plan, 2010; Porter et al., 2012; Seedhouse et al., 2016; Vermeiren et al., 2015) out of 24 mixed-methods studies did not have an Africa-based first or last author, we considered this a key limitation.

Though we did not apply formal thresholds to our ratings of thickness, we identified studies that had been rated ‘effective’ or ‘very effective’ across the different dimensions and considered these as thicker overall. Twelve (Archambault, 2012; Benwell, 2009; Esson et al., 2016; Gough, 2008; Langevang and Gough, 2009; Porter et al., 2010a, 2012, 2017; van Blerk, 2013; Yankson et al., 2017; Zolnikov, 2016; Evans et al., 2018) out of 44 studies containing qualitative data provided thicker description, with mixed-methods studies tending to provide thinner description than pure qualitative studies.

Studies containing quantitative data usually provided clear aims and sufficient detail of participant sampling and recruitment. Studies mainly used self-report (one study (Integrated Transport Plan, 2010) used traffic counting and a further six (Agyemang, 2015; Andreasen and Møller-Jensen, 2017; Evans et al., 2018; Janusz et al., 2019; Kola et al., 2012; Saddier et al., 2016) used mapping or other geographical techniques). Though self-report has known recall biases, this was often the most (or only) feasible way to assess outcomes such as usual travel behaviour. All but three (Diaz Olvera et al., 2016; Schuyler et al., 2017; Venter et al., 2011) quantitative studies were cross-sectional, the vast majority employed simple descriptive analyses that did not account for confounding (with some exceptions (Acheampong and Siiba, 2018; Bartels et al., 2016; Guthold et al., 2011; Guwatudde et al., 2016; John et al., 2017; Joshi et al., 2014; Luke et al., 2014; Nigatu Haregu et al., 2016; Oyeyemi et al., 2011; Oyeyemi et al., 2016; Oyeyemi et al., 2019; Schuyler et al., 2017; Tembe et al., 2018)), and very few made controlled comparisons (with few exceptions (Bartels et al., 2016; Bryceson et al., 2008)). We propose these are the key limitations.

### 3.3 Findings from qualitative data

The mobilities paradigm was the most common theory underpinning qualitative studies (Benwell, 2009; Esson et al., 2016; Gough, 2008; Gwaka, 2018; Langevang and Gough, 2009; Porter et al., 2010a; van Blerk, 2013). Other theories were drawn from sociology (e.g. Kaufmann's concept of mobility and motility (Yankson et al., 2017; Irlam and Zuidgeest, 2018); human geography (e.g. Hägerstrand's time-space framework (Janusz et al., 2019; Vermeiren et al., 2015)); or behavioural sciences (e.g. Theory of Planned Behaviour (El-Dorghamy and Mosa, 2016))). Pure qualitative studies were more likely than mixed-method studies to be explicitly grounded in theory.

The third order constructs are described below.

#### 3.3.1 Mobility as power

We found that physical mobility (i.e. travel) and social mobility were intertwined and inseparable in narratives across sub-Saharan Africa (Archambault, 2012; Esson et al., 2016; Gough, 2008; Langevang and Gough, 2009; Porter et al., 2010b; van Blerk, 2013). Despite a general rhetoric of upwards mobility, participants described trajectories of downward social mobility related to lack of opportunity, particularly in young people (Archambault, 2012; Gough, 2008; Langevang and Gough, 2009; Porter, 2011; Porter et al., 2017). Lack of control over physical mobility reflected a wider lack of power in society (Archambault, 2012; van Blerk, 2013). These power relations were formed through complex legacies of civil war (as in Mozambique), colonialism (Ghana), and in South Africa, apartheid (Archambault, 2012; Benwell, 2009; Esson et al., 2016; Langevang and Gough, 2009). Apartheid entailed formal spatial segregation and marginalisation of ethnic groups. Societal power was enacted through spatial and economic means, resulting in literal and political invisibility of some population segments (Alando and Scheiner, 2016; Benwell, 2009; van Blerk, 2013). Control over mobility was enacted at different levels. Across sub-Saharan Africa there were broader societal influences on mobility, but also within-household factors, such as parents controlling children's mobility (Benwell, 2009; Gough, 2008; Gwaka, 2018; Langevang and Gough, 2009; Porter et al., 2010a, 2010b, 2011, 2012, 2017). Some of these controls become internalised; particularly for girls who aligned their mobility to their expected household roles and perceptions of danger (Benwell, 2009; Porter et al., 2011, 2012).

#### 3.3.2 Travel as an enabler

Travel was viewed as a basic need in order to access food, shelter, education, healthcare, social networks and livelihoods in studies predominantly drawn from Eastern and Western Africa (Gough, 2008; Gwaka, 2018; Langevang and Gough, 2009; Lucas, 2011; Porter, 2011; Porter et al., 2010b; Yankson et al., 2017; Andreasen and Møller-Jensen, 2017; Diaz Olvera et al., 2013; Janusz et al., 2019; Oviedo et al., 2017; Vermeiren et al., 2015). In particular, the transport system was closely interwoven with the economy, livelihoods and employment in these regions (Alando and Scheiner, 2016; Gwaka, 2018; Kamuhanda and Schmidt, 2009; Langevang and Gough, 2009; Lucas, 2011; Porter, 2011; Porter et al., 2010b; Yankson et al., 2017; Evans et al., 2018; Janusz et al., 2019). We noted a disconfirming aspect to this construct; in many instances, travel costs were prohibitive, and these costs coupled with the need to travel could threaten survival activities such as buying food (Alando and Scheiner, 2016; Lucas, 2011; Diaz Olvera et al., 2013; Janusz et al., 2019; Seedhouse et al., 2016; Vermeiren et al., 2015). For the working poor, travel costs could consume the majority of their earnings, resulting in almost net zero economic gain, opportunity cost, and stifled life chances (Lucas, 2011).

#### 3.3.3 Frustrated mobility

We noted recurring narratives of stasis and ennui in sub-Saharan Africa – being stuck, having nowhere to go, and being left behind by a fast moving world. Participants described physical, financial and security barriers to mobility (Archambault, 2012; Gough, 2008; Langevang and Gough, 2009; Kumar, 2011; Porter et al., 2017). Thwarted mobility aspirations were evident among young men in particular, who expressed a longing for travel to far-flung places, to seek adventure, success and status, though this related to longer distance or infrequent travel (Archambault, 2012; Gough, 2008; Langevang and Gough, 2009; Porter et al., 2010a). To compensate, mobile phones have emerged as an important substitute for physical travel across sub-Saharan Africa, but in Eastern Africa in particular (Archambault, 2012; Esson et al., 2016; Gwaka, 2018; Porter, 2011; Porter et al., 2017). In some instances, immobility was desirable, such as traders using other people to deliver goods to customers (Esson et al., 2016; Diaz Olvera et al., 2010; Evans et al., 2018). However, this does not remove the need for travel, but shifts it down the socioeconomic strata to workers in the informal sector (Esson et al., 2016).

#### 3.3.4 Frustrating mobility

Paradoxically, compelled hypermobility occurred alongside immobility. When participants were able to travel, they described having to cover long distances in order to access resources, opportunities and valued destinations, often in hazardous environments, using indirect routes and slow or unreliable modes of transport (Gough, 2008; Gwaka, 2018; Lucas, 2011; Turner and Adzigbey, 2012). This was described particularly in Eastern Africa. We also noted the related phenomena of so-called ‘undesirable’ elements, such as street youth, being forcibly moved on or excluded from public spaces (van Blerk, 2013).

#### 3.3.5 Gendered travel

Travel was related to gendered cultural, religious and social expectations across sub-Saharan Africa. Women were idealised as predominantly immobile homemakers who shaped their travel around household needs, whereas men were idealised as mobile breadwinners (Archambault, 2012; Gough, 2008; Gwaka, 2018; Langevang and Gough, 2009; Porter, 2011; Porter et al., 2010a, 2010b, 2012, 2017; van Blerk, 2013; Amoako-Sakyi and Owusu, 2011; Diaz Olvera et al., 2013). We noted a moral element to travel in young people, particularly young women. Travel was seen as temptation – of promiscuity in young women, and for young men, being drawn into the wrong crowd (including gangsterism, crime and substance abuse). At the same time, travellers in sub-Saharan Africa were seen as potentially powerless victims of mugging, or for young women, sexual violence (Gough, 2008; Gwaka, 2018; Langevang and Gough, 2009; Lucas, 2011; Porter, 2011; Porter et al., 2010a, 2010b, 2012, 2017). Gendered stereotypes across the continent dictated that some modes of travel were inappropriate for women, especially cycling (El-Dorghamy and Mosa, 2016; Porter et al., 2010b, 2011, 2013; Amoako-Sakyi and Owusu, 2011; Seedhouse et al., 2016), but also driving a motorcycle, though riding as a passenger was often considered acceptable (Porter, 2011; Evans et al., 2018; Seedhouse et al., 2016). A study in Mozambique related the lack of employment options for men and thus difficulty attaining the mobile breadwinner ideal to a wider African crisis of masculinity (Archambault, 2012).

#### 3.3.6 Multi-modal mobility

Motorised transport was seen as a need in cities due to long distances (Lucas, 2011). In sub-Saharan Africa, these distances were due to urban sprawl and the positioning of many residential areas (especially unplanned newer areas) far from valued destinations like workplaces, educational facilities and markets (Lucas, 2011; Yankson et al., 2017; Andreasen and Møller-Jensen, 2017; Diaz Olvera et al., 2013; Vermeiren et al., 2015). For the majority, paratransit (informal public transport offered by small-scale private operators including minibus and motorcycle taxis) was the only motorised option available, with wide availability across the continent (El-Dorghamy and Mosa, 2016; Kamuhanda and Schmidt, 2009; Lucas, 2011; Irlam and Zuidgeest, 2018; Kola et al., 2012; Porter et al., 2017). In sub-Saharan Africa, paratransit was seen as flexible and accessible, but had core problems related to road safety and crime, particularly for women (Kamuhanda and Schmidt, 2009; Langevang and Gough, 2009; Lucas, 2011; Raynor, 1923; Diaz Olvera et al., 2010; Evans et al., 2018; Kola et al., 2012; Kumar, 2011; Mbara, 2016; Oviedo et al., 2017; Porter et al., 2017). Across the continent, the spatial reach and availability of formal public transport systems and infrastructure, particularly in low income areas, was described as inadequate (El-Dorghamy and Mosa, 2016; Esson et al., 2016; Lesteven and Boutueil, 2018; Lucas, 2011; Poku-Boansi and Cobbinah, 2018; Porter, 2011; Turner and Adzigbey, 2012; Yankson et al., 2017; Agyemang, 2015; Andreasen and Møller-Jensen, 2017; Delatte et al., 2018; Diaz Olvera et al., 2013; Janusz et al., 2019; Kola et al., 2012; Seedhouse et al., 2016). Private car use was low but increasing (El-Dorghamy and Mosa, 2016), and cars were seen as a badge of success (Delatte et al., 2018), despite car users contributing to high traffic congestion (Andreasen and Møller-Jensen, 2017). We noted enduring stigmas around cycling across the continent, seen as a children's pastime, an indicator of poverty, and inappropriate for women (El-Dorghamy and Mosa, 2016; Porter et al., 2010b, 2011, 2013; Amoako-Sakyi and Owusu, 2011; Andreasen and Møller-Jensen, 2017; Irlam and Zuidgeest, 2018; Nkurunziza et al., 2010; Seedhouse et al., 2016).

#### 3.3.7 Captive walking and walkability

Across the continent, walking was a common mode of transport, but was a ‘captive mode’ undertaken over long distances in hazardous environments, resulting in exertion, opportunity cost and risks to personal safety (El-Dorghamy and Mosa, 2016; Gough, 2008; Gwaka, 2018; Langevang and Gough, 2009; Lesteven and Boutueil, 2018; Lucas, 2011; Poku-Boansi and Cobbinah, 2018; Porter, 2011; Porter et al., 2010a, 2010b, 2011, 2012, 2013, 2017; Delatte et al., 2018; Diaz Olvera et al., 2013; Janusz et al., 2019; Integrated Transport Plan, 2010; Seedhouse et al., 2016). Across the continent, and particularly in South Africa, safety concerns were most salient for women, children and the elderly, leading to mobility restrictions or strategies such as walking in groups, being escorted or not walking at night (Benwell, 2009; Gough, 2008; Gwaka, 2018; Langevang and Gough, 2009; Lucas, 2011; Porter et al., 2010a, 2010b, 2011, 2012, 2013, 2017; Delatte et al., 2018; Oviedo et al., 2017). Women and children also engaged in load-carrying in rural areas of sub-Saharan Africa (e.g. collecting firewood on foot), with attendant issues related to physical exertion, injury and personal safety (Gwaka, 2018; Porter et al., 2010b, 2012, 2013; Zolnikov, 2016; Seedhouse et al., 2016).

#### 3.3.8 Power, policy and planning

Legacies of civil war, colonialism and apartheid (in South Africa), policies built on discriminatory planning schemes, and rapid urbanisation interacted to shape the contemporary form of African cities (Poku-Boansi and Cobbinah, 2018; Yankson et al., 2017). In sub-Saharan Africa, the effects of these combined forces were seen on land use, urban design and transport infrastructure, entrenching spatial inequalities (Benwell, 2009; Lesteven and Boutueil, 2018; Poku-Boansi and Cobbinah, 2018; Yankson et al., 2017). Across the continent, a particular feature of cities described was the informality (and thus lack of regulation) of housing, employment, financial transactions and transport (El-Dorghamy and Mosa, 2016; Esson et al., 2016; Langevang and Gough, 2009; Lesteven and Boutueil, 2018; Poku-Boansi and Cobbinah, 2018; Yankson et al., 2017; Evans et al., 2018; Kola et al., 2012). Many African cities are monocentric, with formal economic activities concentrated in the centre (Lesteven and Boutueil, 2018; Andreasen and Møller-Jensen, 2017) but with informality (particularly residential) on the fringes (Yankson et al., 2017). Tension between formal and informal public transport systems was apparent, most obviously played out through the interaction between Bus Rapid Transit and paratransit in major cities in Eastern, Western and Southern Africa (Lesteven and Boutueil, 2018; Agyemang, 2015; Evans et al., 2018; Seedhouse et al., 2016). In some cities, efforts have been made to align and integrate formal and informal transport systems. In many others, paratransit operates largely outside of formal structures and often within an explicitly anti-paratransit policy context (for example, the exclusion of paratransit from some areas) (Lesteven and Boutueil, 2018; Agyemang, 2015; Evans et al., 2018; Janusz et al., 2019). Absent, fragmented or uncoordinated policy and planning was common in sub-Saharan Africa, with poor public participation overall and for disadvantaged groups (Poku-Boansi and Cobbinah, 2018; Agyemang, 2015; Janusz et al., 2019; Kola et al., 2012; Mbara, 2016; Oviedo et al., 2017; Seedhouse et al., 2016; Vermeiren et al., 2015). This policy vacuum seemed to underlie the current inadequate provision of formal public transport and the rise of paratransit to fill this lacuna in many African cities (Lesteven and Boutueil, 2018; Lucas, 2011; Agyemang, 2015; Oviedo et al., 2017). A modernist agenda for transport planning was described. This focussed on motorised transport including private car use, making non-motorised modes like walking and cycling invisible (Alando and Scheiner, 2016; Evans et al., 2018). Direct and indirect global influences were apparent. Political leaders, particularly those in wealthier African countries, had aspirations for international, ‘future’ cities (Alando and Scheiner, 2016; Lesteven and Boutueil, 2018; van Blerk, 2013; Yankson et al., 2017; Evans et al., 2018; Janusz et al., 2019) through new economies based on gas and oil. At the same time, there was considerable outside investment in Eastern and Southern African roads and public transport systems from China and Europe (Lesteven and Boutueil, 2018; Janusz et al., 2019).

### 3.4 Generation of patterns of hypothesised associations

From each third order construct, we derived predictions that could be interrogated in the quantitative data. We compiled these into a pattern of hypothesised associations for socioeconomic status and gender separately (Table 2). The exception was the third order construct of power, policy and planning; this theme served as a contextual backdrop on which to frame patterns of findings.

**Table 2:** Hypothesised patterns of associations from qualitative studies.

	Hypothesis	Third order construct
*Compared to higher SES individuals, lower SES individuals:*		
1	Live in neighbourhoods a greater distance from the CBD	Mobility as power
2	Have higher prevalence of zero travel (i.e. days where no travel is undertaken)	Frustrated mobility
3	Undertake a lower number of trips	Frustrated mobility
4	Have higher total travel time when they do travel	Frustrating mobility
5	Have longer trip distances when they do travel	Frustrating mobility
6	Are more likely to travel by walking, paratransit and bicycle and less likely to travel by private vehicle	Multi-modal mobility Walking and walkability
7	Walk longer distances	Walking and walkability
8	Are more likely to have travel behaviour dictated by cost	Frustrated mobility Travel as enabler Multi-modal mobility
*Compared to men and boys, women and girls:*		
1	Have higher prevalence of zero travel (i.e. days where no travel is undertaken)	Mobility as power Gendered travel
2	Undertake a lower number of trips	Gendered travel
3	Are less likely to travel by car, motorbike (as driver) and bicycle (as operator) and more likely to travel by walking and paratransit	Gendered travel Multi-modal mobility Walking and walkability
4	Are more likely to be concerned by personal safety when travelling	Gendered travel Multi-modal mobility Walking and walkability
5	Are more likely to have their mobility constrained by families and communities (e.g. due to fears of rape, pregnancy, promiscuity)	Mobility as power Gendered travel
6	Are more likely to have their travel dictated by household responsibilities	Gendered travel
7	Are more likely to engage in load-carrying whilst travelling	Walking and walkability

SES – socioeconomic status; CBD – central business district.

### 3.5 Findings from quantitative data

#### 3.5.1 Socioeconomic status and travel patterns

These results address hypotheses 1–5. Six studies provided some evidence (inferred rather than quantified) that lower income households tend to live in peripheral settlements away from resources and opportunities (hypothesis 1) (Andreasen and Møller-Jensen, 2017; Bechstein, 2010; Chakwizira et al., 2018; Sabry et al., 2017; Tembe et al., 2018; Venter et al., 2011). Among poor households, those that lived peripherally faced more travel difficulties than those that lived centrally (Janusz et al., 2019; Venter et al., 2011). We found limited evidence from three studies that lower socioeconomic status individuals were less likely to travel (Salon and Gulyani, 2010; Schuyler et al., 2017; Venter et al., 2011), and some evidence from six studies that they undertake a lower number of trips (Kumar, 2011; Abiola and Ayodeji, 2012; Alade et al., 2013; Bechstein, 2010; Chakwizira et al., 2018; Masaoe et al., 2011) compared with higher socioeconomic status individuals (hypotheses 2 and 3). There was limited and mixed evidence that total travel time differed by socioeconomic status (hypothesis 4). One study indicated higher travel times in lower socioeconomic groups (Chakwizira et al., 2018), another no difference (Venter et al., 2011) and two reported higher travel time in higher socioeconomic groups (Vermeiren et al., 2015; Alade et al., 2013). We found limited and mostly (but not entirely (Vermeiren et al., 2015) consistent evidence that trip distances were longer for low socioeconomic status individuals in urban areas from three studies (hypothesis 5) (Chakwizira et al., 2018; Sabry et al., 2017; Venter et al., 2011).

#### 3.5.2 Socioeconomic status and mode use

These results address hypotheses 6 and 7. Thirty-three studies provided compelling and consistent evidence that lower socioeconomic status individuals are more likely to travel by walking, paratransit and bike and less likely to travel by private vehicle (hypothesis 6) (Diaz Olvera et al., 2013; Diaz Olvera et al., 2010; Evans et al., 2018; Kola et al., 2012; Kumar, 2011; Oviedo et al., 2017; Integrated Transport Plan, 2010; Porter et al., 2013; Acheampong and Siiba, 2018; Agyemang, 2017; Alade et al., 2013; Amoh-Gyimah and Aidoo, 2013; Bartels et al., 2016; Chakwizira et al., 2018; Diaz Olvera et al., 2016; Elfiky, 2010; Gradidge et al., 2014; Groot and Muthuri, 2017; Kolbe-Alexander et al., 2015; Laverty et al., 2015; Masaoe et al., 2011; Nigatu Haregu et al., 2016; Olawole, 2015; Olojede et al., 2017; Oyeyemi et al., 2016; Oyeyemi et al., 2019; Sabry et al., 2017; Saddier et al., 2016; Salau, 2015; Salon and Aligula, 2012; Salon and Gulyani, 2010; Tembe et al., 2018; Venter et al., 2011). In particular, the quantitative research supported the notion that walking and paratransit are the primary non-motorised and motorised modes of transport, respectively, for the urban working poor (Diaz Olvera et al., 2013; Diaz Olvera et al., 2010; Evans et al., 2018; Kola et al., 2012; Kumar, 2011; Oviedo et al., 2017; Integrated Transport Plan, 2010; Aworemi et al., 2008; Elfiky, 2010; Nigatu Haregu et al., 2016; Salon and Gulyani, 2010; Tembe et al., 2018; Venter et al., 2011). Walking was a common (often the most prevalent) mode of transport used by lower socioeconomic households and individuals, in both rural and urban areas (Diaz Olvera et al., 2013; Kola et al., 2012; Kumar, 2011; Oviedo et al., 2017; Integrated Transport Plan, 2010; Porter et al., 2013; Alade et al., 2013; Bartels et al., 2016; Bryceson et al., 2008; Chakwizira et al., 2018; Diaz Olvera et al., 2016; Elfiky, 2010; Gradidge et al., 2014; Groot and Muthuri, 2017; Kolbe-Alexander et al., 2015; Masaoe et al., 2011; Nigatu Haregu et al., 2016; Olojede et al., 2017; Oyeyemi et al., 2016; Oyeyemi et al., 2019; Salon and Aligula, 2012; Salon and Gulyani, 2010; Venter et al., 2011). As in the qualitative studies, walking was a ‘captive mode’ for those without other options. Limited evidence from three studies suggested that walking distances were longer in lower socioeconomic status individuals (hypothesis 7) (Chakwizira et al., 2018; Salon and Aligula, 2012; Salon and Gulyani, 2010). We saw some nuance in paratransit use by socioeconomic status. Individuals with the highest absolute levels of poverty tended not to use paratransit as they could not afford it, or lived in areas without paved roads that precluded access to most forms of paratransit (Sabry et al., 2017). Conversely, paratransit may be used in some places or situations by even the wealthiest (Integrated Transport Plan, 2010). Studies suggested a substitutional relationship between walking and paratransit in low socioeconomic individuals (Olawole and Olapoju, 2016; Salon and Aligula, 2012; Salon and Gulyani, 2010; Venter et al., 2011). As travel distance increased, paratransit would be used for trips that would otherwise be made by walking if there was sufficient means to pay a paratransit fare. Formal public transport use was low and its availability seen as insufficient (Chakwizira et al., 2018; Tembe et al., 2018; Venter et al., 2011).

Private car ownership and use was confined to a wealthy minority (Kumar, 2011; Agyemang, 2017; Alade et al., 2013; Chakwizira et al., 2018; Kolbe-Alexander et al., 2015; Masaoe et al., 2011; Sabry et al., 2017; Salau, 2015; Salon and Aligula, 2012; Venter et al., 2011). Although cycling was uncommon (Elfiky, 2010; Sabry et al., 2017; Salon and Gulyani, 2010; Venter et al., 2011) (with a few notable exceptions (Bogale, 2012; Diaz Olvera et al., 2013)), we did find evidence that bicycle use was higher in lower socioeconomic individuals (Integrated Transport Plan, 2010; Acheampong and Siiba, 2018; Bechstein, 2010; Elfiky, 2010; Nkurunziza and van Maarseveen, 2013), though with some evidence that recreational cycling was gaining popularity among higher income population segments (Elfiky, 2010).

#### 3.5.3 Socioeconomic status and transport cost

These results address hypothesis 8. We found strong evidence from 13 studies that travel behaviour is dictated by cost in low socioeconomic status individuals (hypothesis 8) (Diaz Olvera et al., 2013; Janusz et al., 2019; Kumar, 2011; Integrated Transport Plan, 2010; Aworemi et al., 2008; Bryceson et al., 2008; Chakwizira et al., 2018; Groot and Muthuri, 2017; Nigatu Haregu et al., 2016; Olojede et al., 2017; Salon and Aligula, 2012; Salon and Gulyani, 2010; Venter et al., 2011). Low income households spend a higher share of household income on transport, often to the extent that this competes with basic needs such as food (Diaz Olvera et al., 2013; Vermeiren et al., 2015; Aworemi et al., 2008; Groot and Muthuri, 2017; Salon and Gulyani, 2010; Venter, 2011; Venter et al., 2011). Transport cost or affordability was typically rated as the strongest influence on mode choice by low socioeconomic status individuals (Integrated Transport Plan, 2010; Aworemi et al., 2008; Chakwizira et al., 2018; Muhammed, 2011; Salon and Aligula, 2012; Salon and Gulyani, 2010; Venter et al., 2011). For paratransit, cost constraints may promote the use of different types of paratransit (e.g. multi passenger vs single passenger modes) (Kumar, 2011), or using paratransit in different ways, such as using paratransit to transport goods but not for personal travel (Evans et al., 2018; Janusz et al., 2019).

We identified several new aspects to travel costs from the quantitative literature. The first was the phenomenon of travelling to seek casual work. If using motorised transport, this entailed a financial outlay that may not be compensated with income (Venter et al., 2011). We also found evidence that low socioeconomic households may own a private vehicle, but not be able to afford to maintain it (Aworemi et al., 2008).

#### 3.5.4 Gender and travel patterns

These results address hypotheses 1 and 2. We found limited evidence from three studies that women or girls have a higher prevalence of zero travel (hypothesis 1) or undertake a lower number of trips (hypothesis 2) (Adetunji, 2012; Odufuwa, 2008; Schuyler et al., 2017).

#### 3.5.5 Gender and mode use

These results address hypothesis 3. We found compelling and consistent evidence from 15 studies that women and girls are less likely to travel by car, motorbike (as driver) and bicycle (as operator) and more likely to travel by walking and paratransit (hypothesis 3) (Diaz Olvera et al., 2013; Diaz Olvera et al., 2010; Evans et al., 2018; Adetunji, 2012; Agyemang, 2017; Amoh-Gyimah and Aidoo, 2013; Babinard, 2011; Bechstein, 2010; Diaz Olvera et al., 2016; Elfiky, 2010; Muhammed, 2011; Olawole, 2015; Sabry et al., 2017; Salon and Gulyani, 2010; Venter et al., 2011). Women were more likely to travel by public transport, and in particular paratransit, whereas men were more likely to travel by private car (Evans et al., 2018; Agyemang, 2017; Amoh-Gyimah and Aidoo, 2013; Diaz Olvera et al., 2016; Sabry et al., 2017; Venter et al., 2011). However, when considering the different types of formal and informal motorised public transport, there were no consistent differences in use between men and women (Abane, 2011; Mbara, 2016; Ojo et al., 2014; Machado-León et al., 2017; Odufuwa et al., 2012). Cycling was low or non-existent in women and to a lesser degree, in girls (Amoako-Sakyi and Owusu, 2011; Bogale, 2012; Irlam and Zuidgeest, 2018; Nkurunziza et al., 2010; Porter et al., 2011; Porter et al., 2013; Acheampong and Siiba, 2018; Bechstein, 2010; Bwire, 2011; Elfiky, 2010; Nkurunziza and van Maarseveen, 2013), and was seen as an inappropriate activity for women (Porter et al., 2012; Porter et al., 2011; Elfiky, 2010). Transport-related physical activity (i.e. walking and cycling together) was higher for men than women (Alemu et al., 2011; Bartels et al., 2016; Dugas et al., 2014; El-Sherbiny and Elsary, 2018; Groot and Muthuri, 2017; Guthold et al., 2011; Guwatudde et al., 2016; John et al., 2017; Joshi et al., 2014; Laverty et al., 2015; Luke et al., 2014; Nigatu Haregu et al., 2016; Oyeyemi et al., 2016; Oyeyemi et al., 2019). Gendered differences in mode use widened with age (Nkurunziza and van Maarseveen, 2013; Salon and Gulyani, 2010).

#### 3.5.6 Gender and mobility constraints

These results address hypotheses 4–7. We found strong evidence from 11 studies that women and girls are perceived as, or perceive themselves as, vulnerable to personal injury and violence (particularly sexual violence) when travelling (hypothesis 4). These safety concerns were particularly salient at night and when walking and using paratransit and public transport (Agyemang, 2015; Delatte et al., 2018; Kumar, 2011; Oviedo et al., 2017; Integrated Transport Plan, 2010; Porter et al., 2017; Babinard, 2011; Behrens and Schalekamp, 2010; Bwire, 2011; Chakwizira et al., 2018; Mfinanga, 2014). However, some (Agyemang, 2015; Integrated Transport Plan, 2010; Oyeyemi et al., 2011) (but not all (Oyeyemi et al., 2019)) studies indicated no association between safety concerns and travel behaviour, possibly due to a lack of feasible alternatives.

We found clear evidence from six studies that mobility constraints were imposed on young women and girls because of concerns about attack, promiscuity and unwanted pregnancy (hypothesis 5) (Porter et al., 2017; Porter et al., 2011; Babinard, 2011; Bwire, 2011; Chakwizira et al., 2018; Schuyler et al., 2017). As seen in the qualitative literature, escorted or group travel was used as a strategy to promote safety (and discourage promiscuity) when travelling (Porter et al., 2017; Porter et al., 2011; Odufuwa, 2008). We found also found clear evidence from seven studies that women's travel was dictated by their household responsibilities (hypothesis 6). This was apparent in two ways: first, that trip purposes tended to be related to household duties (such as shopping and escorting children); and second, that women were more likely to undertake predominantly local travel (Abane, 2011; Janusz et al., 2019; Babinard, 2011; Bartels et al., 2016; Odufuwa et al., 2012; Schuyler et al., 2017; Venter et al., 2011). Limited evidence from two studies indicated that load-carrying related to domestic tasks was more often undertaken by women and girls, particularly in rural areas (hypothesis 7) (Porter et al., 2012; Porter et al., 2013).

### 3.6 Pattern matching

**Fig. 3:**
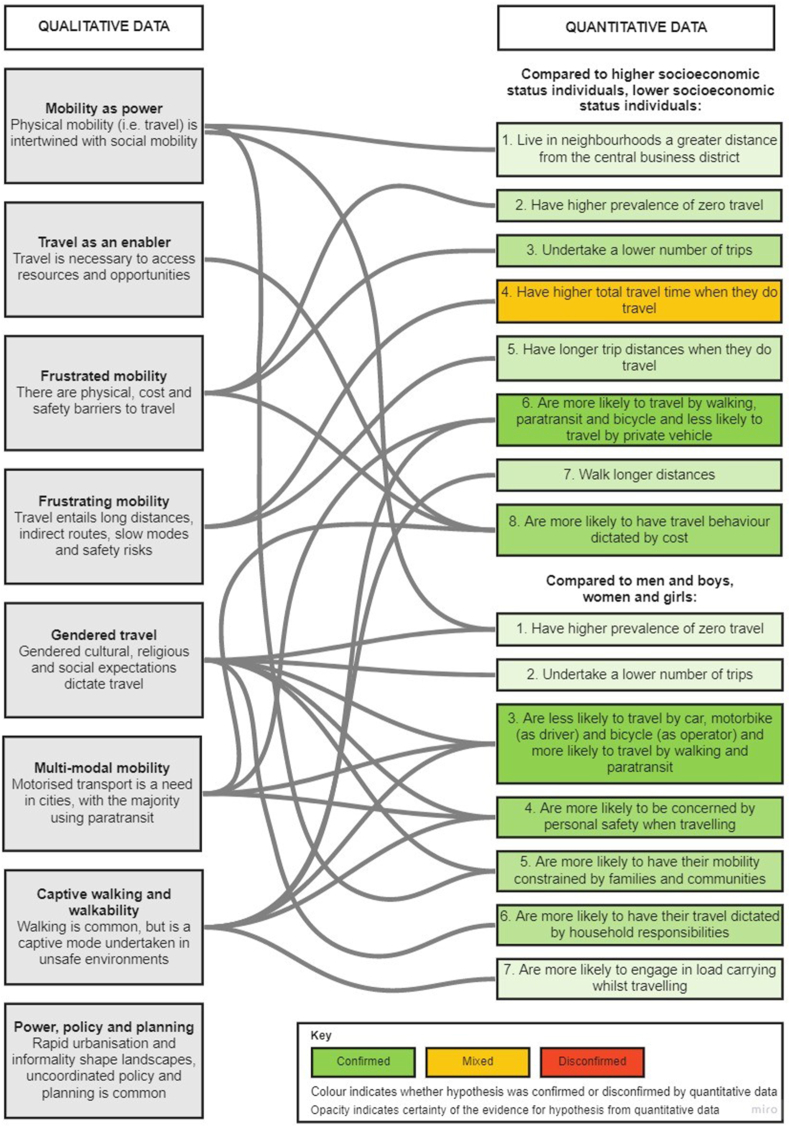
Exploratory sequential analysis and pattern matching. Created using https://miro.com.

The analysis is displayed visually in Fig. 3. In addition to exploring whether and how the literature supported each hypothesis comprising the pattern, we explored how the hypotheses co-occurred within studies, giving further insight into how the literature supported the pattern as a whole.

For socioeconomic status, the overall observed pattern of findings aligned with the hypothesised pattern. Evidence was sparse for some hypotheses, but findings were mostly confirmatory with disconfirmatory evidence found for only one hypothesis. We found that hypotheses 6 (mode use) and 8 (cost dictating travel) frequently co-occurred within studies, implying that cost is a particularly strong driver of mode use rather than travel patterns more generally. We also found that hypotheses 4 (travel time) and 5 (trip distance) frequently co-occurred with hypothesis 6 (mode use), which is consistent with the notion of a relationship between mode used and these parameters.

For gender, a sparser but still confirmatory observed pattern emerged. Our hypothesis on patterns of mode use was mostly confirmed, as were our hypotheses on different types of constraints on travel in women and girls identified from the qualitative literature. We found that hypothesis 3 (mode use) frequently co-occurred with hypothesis 4 (safety concerns) and to a lesser degree with hypothesis 6 (household responsibilities). This highlights the practical constraints on everyday travel, and suggests that safety concerns may be particularly salient for the modes typically used by women and girls.

## 4 Discussion

### 4.1 Main findings

Travel inequity has developed between socioeconomic groups through complex legacies related to civil war, colonialism, and (in South Africa) apartheid. Spatially, this is expressed through sprawling cities with peripheral informal settlements. For poor households, there is a need to traverse large distances and a lack of formal transport infrastructure, with paratransit filling this niche. Travel inequity is entrenched by current political realities. This includes a lack of modernising agency but a modernist agenda for transport that typically does not consider walking and paratransit, the modes most often used by lower socioeconomic groups. Frustrated and frustrating mobility hinder the potential for social mobility and perpetuate poverty.

Gendered social expectations are enduring drivers of travel patterns and ultimately gender-based travel inequity that disadvantages women and girls. We recognise the diversity of countries and cultures across the African continent and the lack of representation of many countries in this review. However, in synthesising the literature we found a recurring set of themes. These related to household division of labour and consequent differences in status, and gendered expectations of behaviour that dictated whether individuals travelled and how. Again, these relationships tended to perpetuate inequity.

We propose that the experience of travel inequity is twofold. First, it is a state of moving but going nowhere, where immobility (lack of opportunity) and hypermobility (opportunity cost) co-exist. Second, it is a state of invisibility. The poor, as well as women and girls, tend to be less visible in society, along with their specific travel needs and the modes of transport they use. This contributes to ongoing social exclusion and marginalisation of these groups.

In lower socioeconomic individuals, travel patterns associated with travel inequity include a reliance on walking and paratransit, being unable to travel due to cost constraints, undertaking a lower amount of travel overall, and travelling long distances in hazardous conditions to meet needs. In women and girls, travel inequity manifests as reliance on walking and lack of access to private vehicles, risk of personal violence, societal attempts to constrain mobility due to concerns about promiscuity and pregnancy, and the need to shape travel around household duties.

### 4.2 Comparison with previous literature

Our observation of transport policy fragmentation is supported by a recent review (Loo and Siiba, 2019) indicating that only 12 African countries had an available transport policy, and of these, three (Uganda, Kenya and South Africa) had a standalone active travel policy. Similarly, reviews indicate that walking and cycling are not well considered in transport planning in sub-Saharan Africa (Sietchiping et al., 2012). Paratransit for the most part operates in a policy vacuum, but where recognised is typically limited or banned (Boutueil et al., 2020). The specific needs of disadvantaged populations (low socioeconomic status groups, women and girls) have not been incorporated in transport planning in Africa despite evidence that travel patterns differ among these groups (Porter, 2008; Sietchiping et al., 2012; Priya Uteng and Turner, 2019).

Our finding that cost is a major barrier to travel is supported by a review of household travel expenditure in Sub-Saharan African cities which indicated that transport costs are regressive, form a major component of poor household expenditure, and that regular public transport use is unaffordable for these households (Diaz Olvera et al., 2008). The concerns we identified around hazardous environments and personal safety when travelling, particularly for pedestrians, are borne out by road safety (World Health Organization, 2013) and violent crime (United Nations Office on, 2005) statistics. However, we found limited evidence of an association between safety concerns and travel behaviour, which we hypothesised was due to a lack of alternatives; this is inconsistent with other literature which suggests that safety- and traffic-related features of built environments do influence travel behaviour in LMICs and in Africa (Day, 2018; Malambo et al., 2017; Isiagi et al., 2021).

### 4.3 Strengths and limitations

To our knowledge, this is the only systematic review drawing together qualitative and quantitative research to describe and understand travel inequity in Africa. We followed established evidence synthesis standards (Moher et al., 2009; France et al., 2019; Campbell et al., 2020), drawing on the perspectives of multi-disciplinary, multi-country researchers and stakeholders. Using a pragmatic pluralist approach (Ogilvie et al., 2020) we found that bringing together different traditions in a mixed methods review gave a complementary and holistic insight; however, we acknowledge the health-focussed lens through which this review was conducted, which influenced our interpretation. We acknowledge the review limitations. In using an exploratory sequential analysis, we conducted a targeted synthesis of the quantitative data, meaning that some insights could have been missed. We were also limited to describing whether differences existed, rather than their magnitude, using the quantitative data. We originally intended to include Caribbean evidence in this review; due to a lack of identified studies this aim could not be satisfied. Finally, we did not search the grey literature, which may have resulted in the omission of relevant studies or documents.

We acknowledge the strengths and limitations of the identified literature. The qualitative data gave rich information on the production and experience of travel inequity; however, as a whole, analysis methods were poorly reported and lacked rigour, and ethical issues were not often considered. The quantitative data allowed us to describe differences in travel behaviour at scale across multiple countries. While simple descriptive analyses can be useful to identify where group differences exist, overall, the literature was limited by the lack of longitudinal assessment and consideration of confounding. A reliance on self-reported travel behaviour may have introduced bias if journey features (e.g. distance or purpose) were systematically misreported. Health-focussed studies typically considered walking and cycling together, which may not be appropriate in this context as patterns of use differed markedly. Similarly, a number of quantitative studies did not clearly distinguish between paratransit and formal public transport. All included studies were published in English, two thirds came from three relatively wealthy African countries, and the majority of these from very large urban conurbations, which limits the review generalisability. More than a third of included studies included did not have an Africa-based first or last author. We suggest that within-continent and within-country collaboration is critical for future research.

### 4.4 Implications for policy and practice

In seminal work in South Africa, Coggin (Coggin and Pieterse, 2015) argues that transport, while not a right in itself, is fundamental in order to access and exercise constitutionally guaranteed rights. We suggest that the cornerstone of equitable pro-poor and pro-female transport policy in Africa should be to support walking and paratransit, and in particular the safety and affordability of these modes, in order to achieve SDG 11.2.

Walking is an existing asset of African transport systems. Increasing walking *per se* should not be a policy priority; in fact, reducing the prevalence of long, captive walking trips is desirable. Rather, attention should be focussed in the short-term on improving the safety of the environment in which (captive) walking is undertaken, including the provision of pedestrian infrastructure. Designing and reshaping cities such that local resources are available within a reasonable walking distance is necessary. Both these recommendations intersect transport and urban design and require multi-sectoral action.

We identified elements of pedestrian safety that are typically less considered in transport planning. It is well known that pedestrians are vulnerable to road traffic injuries, but we found that risk of interpersonal (particularly sexual) violence was usually more salient, alongside hazards such as absent or poorly maintained footpaths. These are not independent – individuals may avoid trip-and-fall hazards by walking on the road, thus increasing the risk of being struck by a vehicle, and absent or isolated pedestrian infrastructure may increase vulnerability to attack. Improving safety would have the additional benefit of promoting walking in wealthier (car-owning) population segments that could benefit from the physical activity associated with walking, while reducing reliance on private motorised transport and improving the sustainability of the transport system as a whole.

Paratransit is a core part of the transport system in Africa, and should be framed as such in policy. However, the difficulties regulating the sector have been demonstrated (Lesteven and Boutueil, 2018; Schalekamp and Behrens, 2010). The target of policy should be to retain the advantages of paratransit (in particular, coverage and flexibility) while mitigating the drawbacks (in particular, safety). Consideration should also be given to how the affordability of paratransit and formal public transport can be improved. This could include strategies aimed at transport providers, including subsidies for transport operations with associated service standards, and transport users, including travel discounts.

We propose that promoting cycling should come secondary to improving walkability due to the vastly higher current mode share for walking. Stakeholders involved in promoting cycling in Africa should be cognisant of the marked cultural and social stigmas to overcome before cycling could become the norm in many places, especially for women.

These policy recommendations must be contextually framed. In Africa, this includes marked international involvement in the transport landscape, including aid funding focussed on road building, the automobile industry as a significant contributor to Gross Domestic Product in many African countries, and private investment in public transport infrastructure from China and Europe. Policy should be forward-looking while incorporating current realities and lived experiences. This means considering what interim, short-term steps could lead towards a more long-term ideal of equitable and sustainable transport systems.

### 4.5 Implications for future research and theory

Context-sensitive, multi-disciplinary research exploring the impacts of transport-related interventions, overall and by socioeconomic status and gender, is core to developing the literature on travel inequity in Africa. For example, a recent review of Bus Rapid Transit in Africa, Asia, and Latin America suggests it offers a variety of benefits to lower income groups, but typically does not benefit the poorest due to insufficient spatial coverage and unaffordability (Venter et al., 2017). Future research may also explore how socioeconomic status and gender intersect. For example, Salon (Salon and Gulyani, 2010) found that women were more likely than men to be poor, but the association between income and mobility was asymmetric (i.e. men's mobility benefited more by not being poor). Our finding that escorted travel is an important feature of mobility in women and girls warrants further research, as does further exploration of a causal link (or lack thereof) between safety-related elements of the built environment and travel behaviour in different groups. Future quantitative studies could look at overall patterns of travel in more detail. Specifically, zero and non-zero travel should be explored separately to avoid masking differences between groups in overall trips or travel duration. We have used two-part regression models (Foley et al., 2017) for this purpose previously.

Future research could be used to build out theoretical models of travel behaviour that could be tested and applied in other contexts. This includes further testing of concordance with or divergence from the hypotheses and patterns described here, as well as their implications for the mobility paradigm. Our findings develop suggestions by others using the mobility paradigm (Benwell, 2009) that neither mobility nor immobility are inherently positive or negative states but that volition and power are critical.

## 5 Conclusions

In Africa, travel inequity perpetuates disadvantage in lower socioeconomic groups, and for women and girls. Our interpretation relates predominantly to urban areas, and is framed by the relatively narrow geographical coverage of the extant literature. Proposed solutions focus on improving the safety and affordability of transport, particularly walking. The realisation of sustainable and equitable transport systems in Africa depends on multi-sectoral collaboration and recognition of the unique needs of different population groups.
