# Supplementary material for: Socioeconomic and gendered inequities in travel behaviour in Africa: Mixed-method systematic review and meta-ethnography
Source: Soc Sci Med. 2022 Jan;292:114545. doi: 10.1016/j.socscimed.2021.114545 (PMC8783052; doi:10.1016/j.socscimed.2021.114545)
Supplement: Multimedia component 1 [file mmc1.docx]

**SUPPORTING INFORMATION CAPTIONS**

S1 Appendix: Reporting guidelines

S2 Appendix: Eligible countries

S3 Appendix: Electronic database search strategies for three databases

S4 Appendix: Senior and junior team members as designated for this review

S5 Appendix: Data extraction template

S6 Appendix: Modified CASP Cohort Study Checklist

S7 Appendix: Modified CASP Qualitative Checklist

S8 Appendix: Thickness rating

S9 Appendix: Characteristics of included studies

**SUPPORTING INFORMATION**

**S1 Appendix: Reporting guidelines**

**SAGER guidelines**

| **General principles** | | **Pg reported** |
| --- | --- | --- |
| • Authors should use the terms *sex* and *gender* carefully in order to avoid confusing both terms. | |  |
| • Where the subjects of research comprise organisms capable of differentiation by sex, the research should be designed and conducted in a way that can reveal sex-related differences in the results, even if these were not initially expected. | |  |
| • Where subjects can also be differentiated by gender (shaped by social and cultural circumstances), the research should be conducted similarly at this additional level of distinction. | |  |
| **Recommendations per section of the article** | |  |
| Title and abstract | If only one sex is included in the study, or if the results of the study are to be applied to only one sex or gender, the title and the abstract should specify the sex of animals or any cells, tissues and other material derived from these and the sex and gender of human participants. | 1,4 |
| Introduction | Authors should report, where relevant, whether sex and/or gender differences may be expected. | 6-7 |
| Methods | Authors should report how sex and gender were taken into account in the design of the study, whether they ensured adequate representation of males and females, and justify the reasons for any exclusion of males or females. | 8-9 |
| Results | Where appropriate, data should be routinely presented disaggregated by sex and gender. Sex- and gender-based analyses should be reported regardless of positive or negative outcome. In clinical trials, data on withdrawals and dropouts should also be reported disaggregated by sex. | 17-21,  24-27 |
| Discussion | The potential implications of sex and gender on the study results and analyses should be discussed. If a sex and gender analysis was not conducted, the rationale should be given. Authors should further discuss the implications of the lack of such analysis on the interpretation of the results. | 28,30-32 |

**PRISMA 2020 Checklist**

| **Section and Topic** | **Item #** | **Checklist item** | **Location where item is reported** |
| --- | --- | --- | --- |
| **TITLE** | | |  |
| Title | 1 | Identify the report as a systematic review. | 1 |
| **ABSTRACT** | | |  |
| Abstract | 2 | See the PRISMA 2020 for Abstracts checklist. | 4 |
| **INTRODUCTION** | | |  |
| Rationale | 3 | Describe the rationale for the review in the context of existing knowledge. | 6-7 |
| Objectives | 4 | Provide an explicit statement of the objective(s) or question(s) the review addresses. | 7 |
| **METHODS** | | |  |
| Eligibility criteria | 5 | Specify the inclusion and exclusion criteria for the review and how studies were grouped for the syntheses. | 8-9 |
| Information sources | 6 | Specify all databases, registers, websites, organisations, reference lists and other sources searched or consulted to identify studies. Specify the date when each source was last searched or consulted. | 9-10 |
| Search strategy | 7 | Present the full search strategies for all databases, registers and websites, including any filters and limits used-. | 51-58 |
| Selection process | 8 | Specify the methods used to decide whether a study met the inclusion criteria of the review, including how many reviewers screened each record and each report retrieved, whether they worked independently, and if applicable, details of automation tools used in the process. | 10-11 |
| Data collection process | 9 | Specify the methods used to collect data from reports, including how many reviewers collected data from each report, whether they worked independently, any processes for obtaining or confirming data from study investigators, and if applicable, details of automation tools used in the process. | 10-11 |
| Data items | 10a | List and define all outcomes for which data were sought. Specify whether all results that were compatible with each outcome domain in each study were sought (e.g. for all measures, time points, analyses), and if not, the methods used to decide which results to collect. | 10-11, 60-69 |
|  | 10b | List and define all other variables for which data were sought (e.g. participant and intervention characteristics, funding sources). Describe any assumptions made about any missing or unclear information. | 10-11, 60-69 |
| Study risk of bias assessment | 11 | Specify the methods used to assess risk of bias in the included studies, including details of the tool(s) used, how many reviewers assessed each study and whether they worked independently, and if applicable, details of automation tools used in the process. | 11, 71-77 |
| Effect measures | 12 | Specify for each outcome the effect measure(s) (e.g. risk ratio, mean difference) used in the synthesis or presentation of results. | 11-13 |
| Synthesis methods | 13a | Describe the processes used to decide which studies were eligible for each synthesis (e.g. tabulating the study intervention characteristics and comparing against the planned groups for each synthesis (item #5)). | 11-13 |
|  | 13b | Describe any methods required to prepare the data for presentation or synthesis, such as handling of missing summary statistics, or data conversions. | 11-13 |
|  | 13c | Describe any methods used to tabulate or visually display results of individual studies and syntheses. | 25 |
|  | 13d | Describe any methods used to synthesize results and provide a rationale for the choice(s). If meta-analysis was performed, describe the model(s), method(s) to identify the presence and extent of statistical heterogeneity, and software package(s) used. | 11-13 |
|  | 13e | Describe any methods used to explore possible causes of heterogeneity among study results (e.g. subgroup analysis, meta-regression). | N/A |
|  | 13f | Describe any sensitivity analyses conducted to assess robustness of the synthesized results. | N/A |
| Reporting bias assessment | 14 | Describe any methods used to assess risk of bias due to missing results in a synthesis (arising from reporting biases). | N/A |
| Certainty assessment | 15 | Describe any methods used to assess certainty (or confidence) in the body of evidence for an outcome. | 12 |
| **RESULTS** | | |  |
| Study selection | 16a | Describe the results of the search and selection process, from the number of records identified in the search to the number of studies included in the review, ideally using a flow diagram. | 14 |
|  | 16b | Cite studies that might appear to meet the inclusion criteria, but which were excluded, and explain why they were excluded. | 14 |
| Study characteristics | 17 | Cite each included study and present its characteristics. | 77-95 |
| Risk of bias in studies | 18 | Present assessments of risk of bias for each included study. | 16-17 |
| Results of individual studies | 19 | For all outcomes, present, for each study: (a) summary statistics for each group (where appropriate) and (b) an effect estimate and its precision (e.g. confidence/credible interval), ideally using structured tables or plots. | N/A |
| Results of syntheses | 20a | For each synthesis, briefly summarise the characteristics and risk of bias among contributing studies. | 16-17 |
|  | 20b | Present results of all statistical syntheses conducted. If meta-analysis was done, present for each the summary estimate and its precision (e.g. confidence/credible interval) and measures of statistical heterogeneity. If comparing groups, describe the direction of the effect. | 17-26 |
|  | 20c | Present results of all investigations of possible causes of heterogeneity among study results. | N/A |
|  | 20d | Present results of all sensitivity analyses conducted to assess the robustness of the synthesized results. | N/A |
| Reporting biases | 21 | Present assessments of risk of bias due to missing results (arising from reporting biases) for each synthesis assessed. | N/A |
| Certainty of evidence | 22 | Present assessments of certainty (or confidence) in the body of evidence for each outcome assessed. | 22-25 |
| **DISCUSSION** | | |  |
| Discussion | 23a | Provide a general interpretation of the results in the context of other evidence. | 28-29 |
|  | 23b | Discuss any limitations of the evidence included in the review. | 29-30 |
|  | 23c | Discuss any limitations of the review processes used. | 29-30 |
|  | 23d | Discuss implications of the results for practice, policy, and future research. | 30-32 |
| **OTHER INFORMATION** | | |  |
| Registration and protocol | 24a | Provide registration information for the review, including register name and registration number, or state that the review was not registered. | 4, 8 |
|  | 24b | Indicate where the review protocol can be accessed, or state that a protocol was not prepared. | 8 |
|  | 24c | Describe and explain any amendments to information provided at registration or in the protocol. | 8, 30 |
| Support | 25 | Describe sources of financial or non-financial support for the review, and the role of the funders or sponsors in the review. | 35 |
| Competing interests | 26 | Declare any competing interests of review authors. | 35 |
| Availability of data, code and other materials | 27 | Report which of the following are publicly available and where they can be found: template data collection forms; data extracted from included studies; data used for all analyses; analytic code; any other materials used in the review. | 60-69, 77-95 |

**PRISMA 2020 for Abstracts Checklist**

| **Section and Topic** | **Item #** | **Checklist item** | **Reported (Yes/No)** |
| --- | --- | --- | --- |
| **TITLE** | | |  |
| Title | 1 | Identify the report as a systematic review. | Yes |
| **BACKGROUND** | | |  |
| Objectives | 2 | Provide an explicit statement of the main objective(s) or question(s) the review addresses. | Yes |
| **METHODS** | | |  |
| Eligibility criteria | 3 | Specify the inclusion and exclusion criteria for the review. | Yes |
| Information sources | 4 | Specify the information sources (e.g. databases, registers) used to identify studies and the date when each was last searched. | Yes |
| Risk of bias | 5 | Specify the methods used to assess risk of bias in the included studies. | Yes |
| Synthesis of results | 6 | Specify the methods used to present and synthesise results. | Yes |
| **RESULTS** | | |  |
| Included studies | 7 | Give the total number of included studies and participants and summarise relevant characteristics of studies. | Yes |
| Synthesis of results | 8 | Present results for main outcomes, preferably indicating the number of included studies and participants for each. If meta-analysis was done, report the summary estimate and confidence/credible interval. If comparing groups, indicate the direction of the effect (i.e. which group is favoured). | Yes |
| **DISCUSSION** | | |  |
| Limitations of evidence | 9 | Provide a brief summary of the limitations of the evidence included in the review (e.g. study risk of bias, inconsistency and imprecision). | Yes |
| Interpretation | 10 | Provide a general interpretation of the results and important implications. | Yes |
| **OTHER** | | |  |
| Funding | 11 | Specify the primary source of funding for the review. | Yes |
| Registration | 12 | Provide the register name and registration number. | Yes |

**eMERGe meta-ethnography reporting guidance**

| **No.** | **Criteria Headings** | **Reporting Criteria** | **Pg reported** |
| --- | --- | --- | --- |
| Phase 1—Selecting meta-ethnography and getting started | | |  |
| *Introduction* | | |  |
| 1 | Rationale and context for the meta-ethnography | Describe the gap in research or knowledge to be filled by the meta-ethnography, and the wider context of the meta-ethnography | 6-7 |
| 2 | Aim(s) of the meta-ethnography | Describe the meta-ethnography aim(s) | 7 |
| 3 | Focus of the meta-ethnography | Describe the meta-ethnography review question(s) (or objectives) | 7 |
| 4 | Rationale for using meta-ethnography | Explain why meta-ethnography was considered the most appropriate qualitative synthesis methodology | 8 |
| Phase 2—Deciding what is relevant | | |  |
| *Methods* | | |  |
| 5 | Search strategy | Describe the rationale for the literature search strategy | 8-9 |
| 6 | Search processes | Describe how the literature searching was carried out and by whom | 9-11 |
| 7 | Selecting primary studies | Describe the process of study screening and selection, and who was involved | 9-11 |
| *Findings* | | |  |
| 8 | Outcome of study selection | Describe the results of study searches and screening | 14 |
| Phase 3—Reading included studies | | |  |
| *Methods* | | |  |
| 9 | Reading and data extraction approach | Describe the reading and data extraction method and processes | 10-11 |
| *Findings* | | |  |
| 10 | Presenting characteristics of included studies | Describe characteristics of the included studies | 14-16, 77-95 |
| Phase 4—Determining how studies are related | | |  |
| *Methods* | | |  |
| 11 | Process for determining how studies are related | Describe the methods and processes for determining how the included studies are related: - Which aspects of studies were compared AND - How the studies were compared | 11-12 |
| *Findings* | | |  |
| 12 | Outcome of relating studies | Describe how studies relate to each other | 11-12 |
| Phase 5—Translating studies into one another | | |  |
| *Methods* | | |  |
| 13 | Process of translating studies | Describe the methods of translation**:** - Describe steps taken to preserve the context and meaning of the relationships between concepts within and across studies- Describe how the reciprocal and refutational translations were conducted- Describe how potential alternative interpretations or explanations were considered in the translations | 11-12 |
| *Findings* | | |  |
| 14 | Outcome of translation | Describe the interpretive findings of the translation. | 17-21 |
| Phase 6—Synthesizing translations | | |  |
| *Methods* | | |  |
| 15 | Synthesis process | Describe the methods used to develop overarching concepts (“synthesised translations”)Describe how potential alternative interpretations or explanations were considered in the synthesis | 11-13 |
| *Findings* | | |  |
| 16 | Outcome of synthesis process | Describe the new theory, conceptual framework, model, configuration, or interpretation of data developed from the synthesis | 25-28 |
| Phase 7—Expressing the synthesis | | |  |
| *Discussion* | | |  |
| 17 | Summary of findings | Summarize the main interpretive findings of the translation and synthesis and compare them to existing literature | 28-29 |
| 18 | Strengths, limitations, and reflexivity | Reflect on and describe the strengths and limitations of the synthesis: - Methodological aspects—for example, describe how the synthesis findings were influenced by the nature of the included studies and how the meta-ethnography was conducted.- Reflexivity—for example, the impact of the research team on the synthesis findings | 29-30 |
| 19 | Recommendations and conclusions | Describe the implications of the synthesis | 30-32 |

**SWiM guidelines**

| SWiM reporting item | Item description | Pg reported | Other* |
| --- | --- | --- | --- |
| **Methods** | | | |
| 1 Grouping studies for synthesis | 1a) Provide a description of, and rationale for, the groups used in the synthesis (eg, groupings of populations, interventions, outcomes, study design) | 8-9 |  |
|  | 1b) Detail and provide rationale for any changes made subsequent to the protocol in the groups used in the synthesis | 8, 30 |  |
| 2 Describe the standardised metric and transformation methods used | Describe the standardised metric for each outcome. Explain why the metric(s) was chosen and describe any methods used to transform the intervention effects, as reported in the study, to the standardised metric, citing any methodological guidance consulted | 12 |  |
| 3 Describe the synthesis methods | Describe and justify the methods used to synthesise the effects for each outcome when it was not possible to undertake a meta-analysis of effect estimates | 12 |  |
| 4 Criteria used to prioritise results for summary and synthesis | Where applicable, provide the criteria used, with supporting justification, to select the particular studies, or a particular study, for the main synthesis or to draw conclusions from the synthesis (eg, based on study design, risk of bias assessments, directness in relation to the review question) | 12 |  |
| 5 Investigation of heterogeneity in reported effects | State the method(s) used to examine heterogeneity in reported effects when it was not possible to undertake a meta-analysis of effect estimates and its extensions to investigate heterogeneity | 12 |  |
| 6 Certainty of evidence | Describe the methods used to assess the certainty of the synthesis findings | 12-13 |  |
| 7 Data presentation methods | Describe the graphical and tabular methods used to present the effects (eg, tables, forest plots, harvest plots) | 12-13, 25, 27, 77-95 |  |
|  | Specify key study characteristics (eg, study design, risk of bias) used to order the studies, in the text and any tables or graphs, clearly referencing the studies included |  |  |
| **Results** | | | |
| 8 Reporting results | For each comparison and outcome, provide a description of the synthesised findings and the certainty of the findings. Describe the result in language that is consistent with the question the synthesis addresses, and indicate which studies contribute to the synthesis | 22-26 |  |
| **Discussion** | | | |
| 9 Limitations of the synthesis | Report the limitations of the synthesis methods used and/or the groupings used in the synthesis and how these affect the conclusions that can be drawn in relation to the original review question | 29-30 |  |

PRISMA=Preferred Reporting Items for Systematic Reviews and Meta-Analyses.

* If the information is not provided in the systematic review, give details of where this information is available (eg, protocol, other published papers (provide citation details), or website (provide the URL)).

**S2 Appendix: Eligible countries**

Eligible African countries:

| Algeria | Mali |
| --- | --- |
| Angola | Mauritania |
| Benin | Mauritius |
| Botswana | Mayotte |
| Burkina Faso | Morocco |
| Burundi | Mozambique |
| Cameroon | Namibia |
| Canary Islands | Niger |
| Cape Verde | Nigeria |
| Central African Republic | Republic of the Congo |
| Chad | Réunion |
| Comoros | Rwanda |
| Democratic Republic of the Congo or Zaire | São Tomé and Príncipe |
| Djibouti | Senegal |
| Egypt | Seychelles |
| Equatorial Guinea | Sierra Leone |
| Eritrea | Somalia |
| Ethiopia | South Africa |
| Gabon | South Sudan |
| Gambia | St Helena |
| Ghana | Sudan |
| Guinea | Swaziland |
| Guinea Bissau | Tanzania |
| Ivory Coast or Côte d'Ivoire | Togo |
| Kenya | Tunisia |
| Lesotho | Uganda |
| Liberia | Western Sahara |
| Libya or Jamahiriya | Zimbabwe |
| Madagascar | Zambia |
| Malawi |  |

**S3 Appendix: Electronic database search strategies for three databases**

**Search strategy for MEDLINE database**

Access through Open Athens: MEDLINE - Ovid MEDLINE(R) and Epub Ahead of Print, In-Process & Other Non-Indexed Citations and Daily

Exposure

1. determinants or determinant or associations or association or correlation or correlations or influenc*.mp

2. exp Risk Factors/ or risk factor*.mp

3. exp POLICY/ or policy or policies or legislat* or law or laws.mp

4. exp ENVIRONMENT/ or exp ENVIRONMENT DESIGN/ or environment*.mp

5. exhaust fume* or emission*.mp

6. exp Vehicle Emissions/

7. exp Traffic-Related Pollution/

8. infrastructur* or neighbo?rhood or rural or countryside* or suburban or sub-urban or settlement* or village* or urban or town or towns or city or cities or slum or slums or pollutant* or pollution or polluted or (air adj3 quality) or weather or climate.mp

9. exp SOCIAL ENVIRONMENT/ or exp Culture/ or cultur*.mp

10. social network* or psychosocial*.mp

11. exp Ethnic Groups/

12. age or sex or gender or ethnic*.mp

13. exp Age Factors/

14. exp Sex/ or exp Sex Factors/

15. exp Demography/ or demograph*.mp

16. exp SOCIOECONOMIC FACTORS/ or socioeconomic* or socio-economic*.mp

17. exp Employment/

18. exp Social Networking/

19. education or qualifi* or profession* or unskilled or skilled or income* or wage or wages or salary or salaries or occupation* or job or jobs or employ* or unemploy* or price* or cost*.mp

20. 1 or 2 or 3 or 4 or 5 or 6 or 7 or 8 or 9 or 10 or 11 or 12 or 13 or 14 or 15 or 16 or 17 or 18 or 19

Outcome

21. exp Transportation/ or transport* or travel* or traffic or transit or paratransit or para-transit or commut*.mp

22. exp Transportation/ and mobility.mp

23. "active mode" or "active modes".mp

24. "sedentary mode" or "sedentary modes".mp

25. exp Walking/ or walk* or pedestrian*.mp

26. exp Bicycling/ or bike or bikes or bicycle or bicycling or bicyclist or biking.mp

27. exp MOTOR VEHICLES/ or automobile* or taxi or taxis or truck or trucks.mp

28. subway or subways or underground or metro or train or trains or rail* or tram or trams or bus or buses or busses or minibus* or minivan*.mp

29. motor-cycl* or motorcycl* or motor-bik* or motorbik* or scooter* or moped* or moto or motocycl* or motocicl*.mp

30. rickshaw* or auto-rickshaw* or autorickshaw* or "two-wheeler*".mp

31. boat* or ferry or ferries.mp

32. matatu or tuktuk* or tuk-tuk* or bodaboda* or boda-boda*.mp

33. "International Physical Activity Question?aire*" or IPAQ or "Global Physical Activity Question?aire*" or GPAQ.mp

34. domain adj3 physical activity.mp

35. pattern* adj3 physical activity.mp

36. 21 or 22 or 23 or 24 or 25 or 26 or 27 or 28 or 29 or 30 or 31 or 32 or 33 or 34 or 35

Population

37. Developing Countries/

38. (developing or "less* developed" or "under-developed" or underdeveloped or middle-income or low-income or lower-income or underserved or "under-served" or deprived or poor*) adj (countr* or nation* or population* or world).mp

39. (developing or "less* developed" or "under-developed" or underdeveloped or middle-income or low-income or lower-income or underserved or "under-served" or deprived or poor*) adj (economy or economies).mp

40. low* adj (gdp or gnp or "gross domestic" or "gross national").mp

41. low* adj3 middle adj3 countr*.mp

42. lmic or lmics or "third world" or "lami countr*".mp

43. "transitional countr*".mp

44. Africa or African or Caribbean or "West Indies".mp

45. exp Africa/ or exp Caribbean Region or exp West Indies/

46. Algeria or Angola or Benin or Botswana or "Burkina Faso" or Burundi or Cameroon or "Canary Islands" or "Cape Verde" or Chad or Comoros or Congo or Djibouti or Egypt or "Equatorial Guinea" or Eritrea or Ethiopia or Gabon or Gambia or Ghana or Guinea or Guinea Bissau or "Ivory Coast" or "Cote dIvoire" or Jamahiriya or Kenya or Lesotho or Liberia or Libya or Madagascar or Malawi or Mali or Mauritania or Mauritius or Mayotte or Morocco or Mozambique or Namibia or Niger or Nigeria or Principe or Reunion or Rwanda or "Sao Tome" or Senegal or Seychelles or "Sierra Leone" or Somalia or "St Helena" or Sudan or Swaziland or Tanzania or Togo or Tunisia or Uganda or "Western Sahara" or Zaire or Zambia or Zimbabwe.mp

47. Cuba or Haiti or "Dominican Republic" or "Puerto Rico" or Jamaica or Trinidad or Tobago or Guadeloupe or Martinique or Bahamas or Barbados or "Saint Lucia" or Curacao or Aruba or "Saint Vincent" or Grenadines or "United States Virgin Islands" or Grenada or Antigua or Barbuda or Dominica or "Cayman Islands" or "Saint Kitts" or Nevis or "Sint Maarten" or Turks or "Caicos Islands" or "Saint Martin" or "British Virgin Islands" or "Caribbean Netherlands" or Anguilla or "Saint Barthelemy" or Montserrat or Belize or Bermuda or Guyana or Suriname or "Virgin Islands" or Antilles or Bonnaire or Panama or Caricom.mp

48. 37 or 38 or 39 or 40 or 41 or 42 or 43 or 44 or 45 or 46 or 47

49. 20 and 36 and 48

50. HIV or "human immunodeficiency virus" or tuberculosis or TB or ebola or zika or malaria or pregnancy or pregnant or matern* or obstetric* or abortion or contracept* or midwife or midwives or postnatal or "sexual health" or "reproductive health".mp

51. 49 NOT 50

52. limit 51 to (yr="2008 -Current")

53. limit 52 to (male or female or humans)

**Search strategy for TRID database**

Access through: https://trid.trb.org/:

TRID - CUSTOM FILTERS: between dates 2008 - 2019 (An expanded search was done for each Index Term used)

Exposure

1. (Keywords) determinants or determinant or associations or association or correlation or correlations or influenc*

2. (Keywords) "risk factor*"

3. (Index Term) Policy --> (Keywords) NN:Cra*

4. (Keywords) policy or policies or legislat* or law or laws

5. (Index Term) Environment, Environmental design, Built environment --> (Keywords) NN:J* or NN:Ttn* or NN:Eb*

6. (Keywords) environment*

7. (Keywords) "exhaust fume*" or emission*

8. (Keywords) "vehicle emissions"

9. (Keywords) "traffic-related pollution"

10. (Keywords) infrastructur* or neighbourhood or neighborhood or rural or countryside* or suburban or sub-urban or settlement* or village* or urban or town or towns or city or cities or slum or slums or pollutant* or pollution or polluted or weather or climate

11. (Keywords) "Air quality"

12. (Index Term) Culture (Social sciences) --> (Keywords) NN:Khr*

13.(Keywords) cultur*

14.(Keywords) "social network*" or psychosocial*

15.(Keywords) age or sex or gender or ethnic*

16.(Index Term) Age Groups --> (Keywords) NN:Mb*

17. (Index Term) Ethnic Groups --> (Keywords) NN:Mha*

18.(Index Term) Population --> (Keywords) NN:Khb*

19.(Keywords) demograph*

20.(Index Term) Socioeconomic factors --> (Keywords) NN:Kj*

21.(Keywords) socioeconomic* or socio-economic*

22.(Index Term) Employment --> (Keywords) NN:Kabkp*

23.(Keywords) education or qualifi* or profession* or unskilled or skilled or income* or wage or wages or salary or salaries or occupation* or job or jobs or employ* or unemploy* or price* or cost*

24. 1 or 2 or 3 or 4 or 5 or 6 or 7 or 8 or 9 or 10 or 11 or 12 or 13 or 14 or 15 or 16 or 17 or 18 or 18 or 20 or 21 or 22 or 23

Outcome

25. (Index Term) Transportation --> (Keywords) NN:A

26. (Keywords) transport* or travel* or traffic or transit or paratransit or para-transit or commut*

27. (Index Term) Transportation --> (Keywords) NN:A AND mobility

28. (Keywords) "active mode" or "active modes"

29. (Keywords) "sedentary mode" or "sedentary modes"

30. (Index Term) Walking, Walking distance --> (Keywords) NN:Aexw* or NN:Bxw*

31. (Keywords) walk* or pedestrian*

32. (Index Term) Bicycling --> (Keywords) NN:Aexb*

33. (Keywords) bicycling or cycle or cycles or bike or bikes or bicycle or bicyclist or biking

34. (Index Term) Motor vehicles --> (Keywords) NN:Qbdd*

35. (Keywords) automobile* or taxi or taxis or truck or trucks or car or cars

36. (Keywords) subway or subways or underground or metro or train or trains or rail* or tram or trams or bus or buses or busses or minibus* or minivan*

37. (Keywords) motor-cycl* or motorcycl* or motor-bik* or motorbik* or scooter* or moped* or moto or motocycl* or motocicl*

38. (Keywords) rickshaw* or auto-rickshaw* or autorickshaw* or two-wheeler*

39. (Keywords) boat* or ferry or ferries

40. (Keywords) matatu or tuktuk* or tuk-tuk* or bodaboda* or boda-boda*

41. (Keywords) "International Physical Activity Questionaire*" or IPAQ or "International Physical Activity Questionnaire*" or "Global Physical Activity Questionaire*" or GPAQ or "Global Physical Activity Questionnaire*"

42. (Keywords) domain* and "physical activity"

43. (Keywords) pattern* and "physical activity"

44. 25 or 26 or 27 or 28 or 29 or 30 or 31 or 32 or 33 or 34 or 35 or 36 or 37 or 38 or 39 or 40 or 41 or 42 or 43

Population

45. (Index Term) Developing countries --> (Keywords) NN:Vwad*

46. (Keywords) "developing countr*" or "less* developed countr*" or "under-developed countr*" or "underdeveloped countr*" or "middle-income countr*" or "low-income countr*" or "lower-income countr*" or "underserved countr*" or "under-served countr*" or "deprived countr*" or "poor* countr*"

47. (Keywords) "developing nation*" or "less* developed nation*" or "under-developed nation*" or "underdeveloped nation*" or "middle-income nation*" or "low-income nation*" or "lower-income nation*" or "underserved nation*" or "under-served nation*" or "deprived nation*" or "poor* nation*"

48. (Keywords) "developing population*" or "less* developed population*" or "under-developed population*" or "underdeveloped population*" or "middle-income population*" or "low-income population*" or "lower-income population*" or "underserved population*" or "under-served population*" or "deprived population*" or "poor* population*"

49. (Keywords) "developing world" or "less* developed world" or "under-developed world" or "underdeveloped world" or "middle-income world" or "low-income world" or "lower-income world" or "underserved world" or "under-served world" or "deprived world" or "poor* world"

50. (Keywords) "developing econom*" or "less* developed econom*" or "under-developed econom*" or "underdeveloped econom*" or "middle-income econom*" or "low-income econom*" or "lower-income econom*" or "underserved econom*" or "under-served econom*" or "deprived econom*" or "poor* econom*"

51. (Keywords) "low* gdp"

52. (Keywords) "low* gnp"

53. (Keywords) "low* gross domestic"

54. (Keywords) "low* gross national"

55. (Keywords) "low* middle" and count*

56. (Keywords) lmic or lmics or "third world" or "lami countr*"

57. (Keywords) "transitional countr*"

58. (Keywords) Africa or African or Caribbean or "West Indies"

59. (Keywords) Algeria or Angola or Benin or Botswana or "Burkina Faso" or Burundi or Cameroon or "Canary Islands" or "Cape Verde" or Chad or Comoros or Congo or Djibouti or Egypt or "Equatorial Guinea" or Eritrea or Ethiopia or Gabon or Gambia or Ghana or Guinea or Guinea Bissau or "Ivory Coast" or "Cote dIvoire" or Jamahiriya or Jamahiriya or Kenya or Lesotho or Liberia or Libya or Madagascar or Malawi or Mali or Mauritania or Mauritius or Mayotte or Morocco or Mozambique or Namibia or Niger or Nigeria or Principe or Reunion or Rwanda or "Sao Tome" or Senegal or Seychelles or "Sierra Leone" or Somalia or "St Helena" or Sudan or Swaziland or Tanzania or Togo or Tunisia or Uganda or "Western Sahara" or Zaire or Zambia or Zimbabwe

60. (Keywords) Cuba or Haiti or "Dominican Republic" or "Puerto Rico" or Jamaica or Trinidad or Tobago) or Guadeloupe or Martinique or Bahamas or Barbados or "Saint Lucia" or Curacao or Aruba or "Saint Vincent" or Grenadines or "United States Virgin Islands" or Grenada or Antigua or Barbuda or Dominica or "Cayman Islands" or "Saint Kitts" or Nevis or "Sint Maarten" or Turks or "Caicos Islands" or "Saint Martin" or "British Virgin Islands" or "Caribbean Netherlands" or Anguilla or "Saint Barthelemy" or Montserrat or Belize or Bermuda or Guyana or Suriname or "Virgin Islands" or Antilles or Bonnaire or Panama or Caricom

61. 45 or 46 or 47 or 48 or 49 or 50 or 51 or 52 or 53 or 54 or 55 or 56 or 57 or 58 or 59 or 60

62. HIV or "human immunodeficiency virus" or tuberculosis or TB or ebola or zika or malaria or pregnancy or pregnant or matern* or obstetric* or abortion or contracept* or midwife or midwives or postnatal or "sexual health" or "reproductive health"

63. (24 and 44 and 61) NOT 62

***** no other limits applied *****

**Search strategy for SCOPUS database**

Access through: Cambridge LibGuides

Exposure

1. TITLE-ABS-KEY determinant or association or correlation or influenc*

2. TITLE-ABS-KEY "risk factor"

3. TITLE-ABS-KEY policy or legislat* or law

4. TITLE-ABS-KEY environment*

5. TITLE-ABS-KEY "exhaust fume" or emission

6. TITLE-ABS-KEY "vehicle emission"

7. TITLE-ABS-KEY "traffic-related pollution"

8. TITLE-ABS-KEY infrastructur* or neighborhood or rural or countryside or suburban or sub-urban or settlement or village or urban or town or city or slum or pollutant or pollution or polluted or (air w/3 quality) or weather or climate

9. TITLE-ABS-KEY "social environment" or cultur*

10. TITLE-ABS-KEY "social network*" or psychosocial*

11. TITLE-ABS-KEY age or sex or gender or ethnic*

12. TITLE-ABS-KEY demograph*

13. TITLE-ABS-KEY socioeconomic*

14. TITLE-ABS-KEY education or qualifi* or profession* or unskilled or skilled or income* or wage or salary or occupation* or job or employ* or unemploy* or price* or cost*

15. 1 or 2 or 3 or 4 or 5 or 6 or 7 or 8 or 9 or 10 or 11 or 12 or 13 or 14

Outcome

16. TITLE-ABS-KEY transport* or travel* or traffic or transit or paratransit or commut*

17. TITLE-ABS-KEY transportation AND mobility

18. TITLE-ABS-KEY {active mode} or {active modes}

19. TITLE-ABS-KEY {sedentary mode} or {sedentary modes}

20. TITLE-ABS-KEY walk* or pedestrian*

21. TITLE-ABS-KEY bike or bicycle or bicycling or bicyclist or biking

22. TITLE-ABS-KEY "motor vehicle" or automobile or taxi or truck

23. TITLE-ABS-KEY subway or underground or metro or train or rail* or tram or bus or minibus* or minivan*

24. TITLE-ABS-KEY motor-cycl* or motorcycl* or motor-bik* or motorbik* or scooter* or moped* or moto or motocycl* or motocicl*

25. TITLE-ABS-KEY rickshaw* or auto-rickshaw* or autorickshaw* or two-wheeler*

26. TITLE-ABS-KEY boat* or ferry

27. TITLE-ABS-KEY matatu or tuktuk* or "tuk-tuk*" or bodaboda* or "boda-boda*"

28. TITLE-ABS-KEY "International Physical Activity Questionaire*" or IPAQ or "Global Physical Activity Questionaire*" or GPAQ

29. TITLE-ABS-KEY domain w/3 "physical activity"

30. TITLE-ABS-KEY pattern* w/3 "physical activity"

31. 16 or 17 or 18 or 19 or 20 or 21 or 22 or 23 or 24 or 25 or 26 or 27 or 28 or 29 or 30

Population

32. TITLE-ABS-KEY (developing or "less* developed" or "under developed" or underdeveloped or "middle-income" or "low*-income" or underserved or "under-served" or deprived or poor*) pre/1 (countr* or nation* or population* or world)

33. TITLE-ABS-KEY (developing or "less* developed" or "under developed" or underdeveloped or "middle-income" or "low*-income" or underserved or "under-served" or deprived or poor*) pre/1 (economy or economies)

34. TITLE-ABS-KEY low* pre/1 (gdp or gnp or {gross domestic} or {gross national})

35. TITLE-ABS-KEY low* w/3 middle w/3 countr*

36. TITLE-ABS-KEY lmic or lmics or {third world} or "lami countr*"

37. TITLE-ABS-KEY "transitional countr*"

38. TITLE-ABS-KEY Africa or African or Caribbean or {West Indies}

39. TITLE-ABS-KEY (Algeria or Angola or Benin or Botswana or {Burkina Faso} or Burundi or Cameroon or {Canary Islands} or {Cape Verde} or Chad or Comoros or Congo or Djibouti or Egypt or {Equatorial Guinea} or Eritrea or Ethiopia or Gabon or Gambia or Ghana or Guinea or {Guinea Bissau} or {Ivory Coast} or {Cote dIvoire} or Jamahiriya or Kenya or Lesotho or Liberia or Libya or Madagascar or Malawi or Mali or Mauritania or Mauritius or Mayotte or Morocco or Mozambique or Namibia or Niger or Nigeria or Principe or Reunion or Rwanda or {Sao Tome} or Senegal or Seychelles or {Sierra Leone} or Somalia or {St Helena} or Sudan or Swaziland or Tanzania or Togo or Tunisia or Uganda or {Western Sahara} or Zaire or Zambia or Zimbabwe)

40. TITLE-ABS-KEY (Cuba or Haiti or {Dominican Republic} or {Puerto Rico} or Jamaica or Trinidad or Tobago or Guadeloupe or Martinique or Bahamas or Barbados or {Saint Lucia} or Curacao or Aruba or {Saint Vincent} or Grenadines or {United States Virgin Islands} or Grenada or Antigua or Barbuda or Dominica or {Cayman Islands} or {Saint Kitts} or Nevis or {Sint Maarten} or Turks or {Caicos Islands} or {Saint Martin} or {British Virgin Islands} or {Caribbean Netherlands} or Anguilla or {Saint Barthelemy} or Montserrat or Belize or Bermuda or Guyana or Suriname or {Virgin Islands} or Antilles or Bonnaire or Panama or Caricom)

41. 32 or 33 or 34 or 35 or 36 or 37 or 38 or 30 or 40

42. 15 and 31 and 41

43. 42 AND NOT (TITLE-ABS-KEY (HIV or {human immunodeficiency virus} or tuberculosis or TB or ebola or zika or malaria or pregnancy or pregnant or matern* or obstetric* or abortion or contracept* or midwife or postnatal or {sexual health} or {reproductive health})

44. limit 43 to (yr="2008 -Current")

45. With limit to male or female or humans

**S4 Appendix: Senior and junior team members as designated for this review**

[author details removed for submission]

**S5 Appendix: Data extraction template**

| **Field** | **Standard or custom  Covidence field** | **Format** | **Notes** |
| --- | --- | --- | --- |
| **Identification** |  |  |  |
| Study details |  |  |  |
| Sponsorship source | Standard | Free text | Check whether a funding source is reported. Often this can be found in a section titled ‘Acknowledgements’ or ‘Funding’ or reported on the first page of the manuscript near the author information.  Enter the funding source  If more than one, list separated by a comma  Otherwise enter ‘no source reported’  *Example*  Medical Research Council, Heart Foundation |
| Country | Standard | Free text | Enter the country where the data were collected  If more than one, list separated by a comma  *Example*  Ghana |
| Setting | Standard | Free text | Enter the setting where the data were collected  If more than one, list separated by a comma  If information given at different levels of geographical aggregation (e.g. region, city, district), list all this information and where possible give information on the level of aggregation in brackets  *Example*  Accra, Kumasi, Tamale, Sekondi–Takoradi (metropolitan areas) |
| Comments | Standard | Free text | Leave this field blank or use for general comments/queries arising |
| Author’s contact details |  |  |  |
| Author's name | Standard | Free text | Enter the name of the first author  Use the format [surname, initials]  *Example*  Abane, AB |
| Institution | Standard | Free text | Enter the institution or affiliation of the first author with the country of this affiliation in brackets  If more than one, list separated by a comma  *Example*  University of Cape Coast (Ghana) |
| Email | Standard | Free text | Enter the email address of the first author  If none provided enter ‘email not provided’  *Example*  [am_abane@yahoo.com](mailto:am_abane@yahoo.com) |
| Address | Standard | Free text | Leave this field blank |
| Additional identification data |  |  |  |
| Country - first author | Custom | Free text | Enter the country where the first author’s institution or affiliation is located  If more than one, list the country of the first affiliation  *Example*  Kenya |
| Country - last author | Custom | Free text | Enter the country where the last author’s institution or affiliation is located  If more than one, list the country of the first affiliation  If the study has only one author, enter ‘no last author’  *Example*  United States of America |
| Data source | Custom | Free text  Categorical | Enter the source of the data  Use the following categories:  If more than one, list separated by a comma     - Academic - Research Institute/Centre - Government - Private sector - International organisation - NGO - Other   Academic: research studies led by universities  Research Institute/Centre: research studies led by research groups which are part of a larger institution such as universities, hospitals, corporations, etc.  Government: routine monitoring such as national surveys or traffic monitoring  Private sector: research studies led by industry, private companies, consultancy firms, etc.  International organisation: research studies led by organisations such as WHO, World Bank, FAO, UN, etc.  NGO: research studies led by non-profit, non-governmental organisations  Other: enter other type of source of data not listed above  *Example*  Academic, Research Institute/Centre, International organisation |
| Date - data collection | Custom | Free text | Enter the start and end year of data collection  If data collection was completed within one year, enter the year only  If one discrete period of data collection spanned multiple years, enter the start and end year separated by a dash  If there were multiple discrete periods of data collection (e.g. in a longitudinal cohort that had a baseline and follow-up data collection), enter the years separated by a comma  *Examples*  2014  2014-2015 (one discrete period of data collection)  2014, 2017 (multiple periods of data collection e.g. cohort)  2015-2015, 2017-2018 (multiple periods of data collection, each spanning multiple years) |
| Date - publication | Custom | Free text | Enter the year that the study was published  *Example*  2014 |
| GDAR - work package 1b | Custom | Free text | Enter ‘WP1flag’ if the study uses spatial assessment, or provides information on transportation patterns, physical activity patterns, road traffic accidents, air pollution, demographics or health outcomes  Otherwise enter ‘none’ |
| GDAR - work package 3 | Custom | Free text | Enter ‘WP3flag’ if the study refers to policy in South Africa, Kenya, Cameroon or Jamaica, and provides information on the sector of origin of the policy such as health or transport  Otherwise enter ‘none’ |
| Equity | Custom | Free text | Enter ‘equityflag’ if the study explores gender or sex differences in travel behaviour, or differences by socioeconomic status to inform an equity analysis  Otherwise enter ‘none’ |
| **Methods** |  |  |  |
| Design |  |  | Leave this field blank |
| Additional methods data |  |  |  |
| A. Research type | Standard | Categorical | Enter the research type:  Use ONE of the following categories:   - Quantitative - Qualitative - Mixed method   Quantitative: measures of values or counts expressed as numbers e.g. the data generated from surveys, questionnaires, measurements  Qualitative: description of phenomena often expressed as text e.g. interviews, focus groups  Mixed method: Both quantitative and qualitative methods used |
| B. Study design - quantitative | Custom | Free text  Categorical | Enter the study design.  Quantitative or mixed method study - use ONE of the following categories:   - Randomised controlled trial - Cohort study - Case-control study - Cross sectional study - Case report / Case study - Other – please, specify   For quantitative or mixed method studies, complete this field. Otherwise (i.e. for qualitative studies) enter ‘not applicable’ |
| C. Study design - qualitative | Custom | Free text  Categorical | Enter the study design.  Qualitative or mixed method study – use ONE of the following categories:   - Grounded theory - Case study - Historical / Narratives - Participatory research / Action research - Phenomenology - Ethnography / Observation - Other – please, specify   Ethnography: Immersion of the researcher the participants’ environment, typically through observation (note, do not confuse this design with observational quantitative designs like traffic counting)  For qualitative or mixed method studies, complete this field. Otherwise (i.e. for quantitative studies) enter ‘not applicable’ |
| D. Study method – quantitative | Custom | Free text | If the study includes a quantitative element, enter the method type.  Enter a small amount of free text summarising the study methods. This can usually be cut and pasted from the abstract.  *Examples*  Two-day vox pop survey, structured questionnaire at two main trip destinations  STEPs survey  Household survey and interview  For quantitative or mixed method studies, complete this field. Otherwise (i.e. for qualitative studies) enter ‘not applicable’ |
| E. Study method - qualitative | Custom | Free text  Categorical | If the study includes a qualitative element, enter the method type.  Use the following categories:  If more than one, list separated by a comma     - Structured – interview , survey, questionnaire - Un-structured or Semi structured - interview , survey, questionnaire - In depth- interview / Key informants - Focus groups / Group discussions - Field Notes - Narrative descriptions - Audio tapes - Video tapes - Seasonal calendars - Transect walks - Participatory mapping / Modelling - Other– please, enter a small amount of free text summarising the study methods.   For qualitative or mixed method studies, complete this field. Otherwise (i.e. for quantitative studies) enter ‘not applicable’ |
| F. Exposure category | Custom | Free text  Categorical | Enter ALL of the categories of exposure that were assessed in the study.  Use the following categories:  If more than one, list separated by a comma     - Individual characteristics - Travel mode characteristics - Built environment - Natural environment - Socio-cultural environment - Policy or wider environment   See the ‘Categories of exposure’ (Table 1) in this document to help you decide which exposures should be listed.  Exposures must be linked explicitly with travel behaviour outcomes, not just reported. E.g. A study might report the sex distribution of the sample (i.e. numbers of males and females), but to be considered an exposure the study needs to have examined how travel behaviour varies by sex (e.g. whether public transport use differs between males and females). For this example, the following category should be entered: ‘Individual characteristics’. |
| G. Exposure/s | Custom | Free text | Enter all of the exposures that were assessed in the study. See Table 1 to help you decide how to report exposures. |
| H. Exposure methods | Custom | Free text  Categorical | Enter ONE of the methods of exposure that were assessed in the study:   - Subjective - Objective - Both subjective and objective   Subjective: Usually this means the participant has self-reported  Objective: Observed by the researcher. For example, measured population density using census data, measured land use mix using geographical information systems |
| I. Outcome/s | Custom | Free text  Categorical | Enter ALL of the following outcomes that were assessed in the study:  Enter all outcomes assessed from this list, separated by a comma.     - Time in all travel - Time in active travel - Time in travel modes or combinations of modes - Number of trips/journeys - Use of travel modes - Choice of travel modes - Travel mode share - Mobility budget (percentage) - Purpose of travel mode use - Other – please, specify   Active travel is commonly operationalised as walking only, cycling only, walking and cycling combined, or walking, cycling and public transport combined  Enter all outcomes assessed from this list, separated by a comma  *Example*  Time in all travel, time in active travel |
| J. Outcome methods | Custom | Free text  Categorical | Enter ONE of the methods of outcome that were assessed in the study:   - Subjective - Objective - Both subjective and objective   Subjective: Usually this means the participant has self-reported  Objective: Observed by the researcher. For example, counting people using particular travel modes |
| K. Analysis method - quantitative | Custom | Free text | Enter a small amount of free text summarising the analysis methods. For quantitative studies it is important to distinguish between basic statistical methods for assessing relationships (e.g. correlations) and those that account for potential confounding factors (e.g. multivariable regression modelling).  For quantitative or mixed method studies, complete this field. Otherwise (i.e. for qualitative studies) enter ‘not applicable’ |
| L. Analysis method - qualitative | Custom | Free text | Enter a small amount of free text summarising the analysis methods, e.g. thematic analysis.  For qualitative or mixed method studies, complete this field. Otherwise (i.e. for quantitative studies) enter ‘not applicable’ |
| **Population** |  |  |  |
| Inclusion criteria | Standard | Free text | Enter a small amount of free text summarising the types of participants included in the study. This can usually be cut and pasted from the article.  *Example*  Data were collected from passengers waiting to board or actually on board vehicles operated by privately-owned and government-assisted transport systems in the four metropolitan areas under study. |
| Exclusion criteria | Standard | Free text | Enter a small amount of free text summarising the types of participants excluded from the study. This can usually be cut and pasted from the article.  In many cases, there will be no explicit exclusion criteria listed. If this is the case, enter ‘not reported’.  *Example*  Institutionalised individuals (e.g. hospitalised or jailed) were excluded from the study  Participants with a diagnosis of cancer were excluded |
| Group differences | Standard | Free text | Enter a small amount of free text summarising whether there were any differences between participant groups at baseline. This only applies to studies that used a controlled experimental design (and it is likely that the majority of literature identified will not use this type of design). This can usually be cut and pasted from the article.  If this does not apply, enter ‘not applicable’. |
| Additional population data |  |  |  |
| 1. Sample size | Custom | Free text | Enter a small amount of free text describing the sample size  *Examples*  10,128 individuals  100 households, 1 member of each household  Ouagadougou - 754 households, 3682 individuals, Bamako - 251 households, 1666 individuals |
| 2. Response rate | Custom | Free text | Enter a small amount of free text describing the response rate, if provided. If reported, this is typically expressed as a percentage. It is likely that many studies will not provide this information.  If not reported, enter ‘not reported’  *Example*  78% response rate |
| 3. Age | Custom | Free text | Enter a small amount of free text describing the age of participants. If possible, enter this in the format ‘mean (standard deviation)’. Other common reporting formats are a range, or the proportion of participants that fall within a particular age range.  If not reported, enter ‘not reported’  *Examples*  45.3 (2.1) years  15-60 years  48% of participants were aged 30-44 years  Over 13 years |
| 4. Sex | Custom | Free text | Enter a small amount of free text describing the sex of participants. If possible, enter this in the form ‘number (percentage)’  If not reported, enter ‘not reported’  *Examples*  100 (25%) female; 300 (75%) male  55% female  Female only |
| 5. Urban/rural | Custom | Free text | Enter a small amount of free text describing the urban/rural status of participants  If not reported, enter ‘not reported’  *Examples*  Rural  Urban  Urban and peri-urban dwellers |
| 6. Direction of relationships | Custom | Free text | Enter a small amount of free text summarising the direction of the relationships found between exposures and outcomes. Try to keep this as succinct as possible, although it is likely that some studies will report a large number of relationships. Do not worry about reporting the size of the relationship.  *Examples*  Women travelled less than men  Where new roads were built, car traffic increased |
| 7. Main idea | Custom | Free text | Imagine you would like to summarise the article to a colleague in few sentences. What would you tell him/her?  Try to get the main idea of the article. Limit your answer to 1 paragraph containing 3 to 5 sentences (maximum) |
| Baseline characteristics |  |  |  |
| **Interventions** |  |  | Do not enter data in this section |
| **Outcomes** |  |  | Do not enter data in this section |

**Categories of exposure**

| **Exposure category** | **Examples** |
| --- | --- |
| Individual characteristics | Age  Sex  Ethnicity  Urban vs. rural dweller  Slum dweller  Country, region or area of residence  Socioeconomic status  Household factors (e.g. size, assets)  Employment status  Mobility budget (e.g. the share of income spent on public transport)  Education level  Occupation  Income  Marital status  Vehicle ownership (e.g. car or motorbike)  Bike ownership  Hold driving license  Ability to cycle  Attitude towards particular travel modes  Knowledge about travel modes  Distance needed to travel (e.g. to place of work)  Lifestyle  Health status |
| Travel mode characteristics | Safety (e.g. road traffic accidents, injuries or fatalities)  Crime (e.g. interpersonal violence, harassment, mugging)  Cost  Comfort  Customer service  Accessibility  Availability of services  Availability of space  Speed (e.g. traffic jam, delays)  Security  Price stability |
| Built environment | Population density/urban sprawl  Land use mix  Street connectivity  City layout (e.g. shops or other community destinations all clustered in city centre)  Transport infrastructure (e.g. provision of paved roads, public transport, footpaths/cycling paths or infrastructure that protects vulnerable users)  Sidewalk or street-crossing quality  Cycle path quality  Street lighting  Speed management infrastructure  General levels of traffic  Green space  Pollution or air quality  Aesthetics (e.g. pleasantness of area)  Incivilities (e.g. crime, litter, upkeep of area, traffic noise)  Note: This includes individuals’ perceptions of the built environment as well as objective assessments of the built environment |
| Natural environment | Climate (e.g. rainy vs. dry season, winter vs. summer)  Weather (e.g. hot temperatures)  Natural disasters  Note: This includes individuals’ perceptions of the natural environment as well as objective assessments of the natural environment |
| Socio-cultural environment | Social norms (e.g. family/household member roles, or community values)  Gender roles  Religious norms  Cultural norms  Class segregation  Stigmatisation/aspiration of particular travel modes (e.g. car seen as indicator of wealth) |
| Policy or wider environment | Urban planning (e.g. planned vs. unplanned development)  Regional or national travel budgets or policies (including policy priorities)  Regional or national physical activity policies (including policy priorities)  Transport sector regulation/management, including taxes  Corruption/mismanagement  Economic development (e.g. leading to changing types of occupations)  Legacies (e.g. of apartheid or colonisation)  Climate/sustainability agenda (including oil crisis)  Overseas aid  Globalisation  International policy priorities  War and conflict |

**S6 Appendix: Modified CASP Cohort Study Checklist**

| **#** | **Item** | | **Elaboration** |
| --- | --- | --- | --- |
| Section A: Are the results of the study valid? | | | |
| 1 | Did the study address a clearly focussed issue?  Yes  Can’t tell  No | HINT: A question can be ‘focused’ in terms of:   - the population studied - the risk factors studied - is it clear whether the study tried to detect a beneficial or harmful effect the outcomes considered | |
| 2 | Was the cohort/sample recruited in an acceptable way?  Yes  Can’t tell  No | HINT: Look for selection bias which might compromise the generalisability of the findings:   - was the cohort/sample representative of a defined population - was there something special about the cohort/sample - was everybody included who should have been | |
| 3 | Was the exposure accurately measured to minimise bias?  Yes  Can’t tell  No | HINT: Look for measurement or classification bias:   - did they use subjective or objective measurements - do the measurements truly reflect what you want them to (have they been validated) - were all the subjects classified into exposure groups using the same procedure | |
| 4 | Was the outcome accurately measured to minimise bias?  Yes  Can’t tell  No | HINT: Look for measurement or classification bias:   - did they use subjective or objective measurements - do the measurements truly reflect what you want them to (have they been validated) - has a reliable system been established for detecting all the cases (for measuring disease occurrence) - were the measurement methods similar in the different groups - were the subjects and/or the outcome assessor blinded to exposure (does this matter) | |
| 5a | Have the authors identified all important confounding factors?  Yes  Can’t tell  No | HINT: list the ones you think might be important, and ones the author missed | |
| 5b | Have they taken account of the confounding factors in the design and/or analysis?  Yes  Can’t tell  No | HINT: look for restriction in design, and techniques e.g. modelling, stratified-, regression-, or sensitivity analysis to correct, control or adjust for confounding factors | |
| 6a | For longitudinal studies: Was the follow up of subjects complete enough?  Yes  Can’t tell  No  N/A (for cross-sectional studies) | HINT: Consider:   - the good or bad effects should have had long enough to reveal themselves - the persons that are lost to follow-up may have different outcomes than those available for assessment - in an open or dynamic cohort, was there anything special about the outcome of the people leaving, or the exposure of the people entering the cohort | |
| 6b | For longitudinal studies: Was the follow up of subjects long enough?  Yes  Can’t tell  No  N/A (for cross-sectional studies) | HINT: Consider:   - the good or bad effects should have had long enough to reveal themselves - the persons that are lost to follow-up may have different outcomes than those available for assessment - in an open or dynamic cohort, was there anything special about the outcome of the people leaving, or the exposure of the people entering the cohort | |
| Section B: What are the results? | | | |
| 7 | What are the results of this study?  [free text] | HINT: Consider:   - what are the bottom line results - have they reported the rate or the proportion between the exposed/unexposed, the ratio/rate difference - how strong is the association between exposure and outcome (RR) - what is the absolute risk reduction (ARR) | |
| 8 | How precise are the results?  [free text] | HINT: look for the range of the confidence intervals, if given | |
| 9 | Do you believe the results?  Yes  Can’t tell  No | HINT: Consider:   - big effect is hard to ignore - can it be due to bias, chance or confounding - are the design and methods of this study sufficiently flawed to make the results unreliable - Bradford Hills criteria (e.g. time sequence, dose-response gradient, biological plausibility, consistency) | |
| Section C: Will the results help locally? | | | |
| 10 | Can the results be applied to the local population?  Yes  Can’t tell  No | HINT: Consider whether:   - a cohort/cross-sectional study was the appropriate method to answer this question - the subjects covered in this study could be sufficiently different from your population to cause concern - your local setting is likely to differ much from that of the study - you can quantify the local benefits and harms | |
| 11 | Do the results of this study fit with other available evidence?  Yes  Can’t tell  No |  | |
| 12 | What are the implications of this study for practice?  Yes  Can’t tell  No | HINT: Consider:   - one observational study rarely provides sufficiently robust evidence to recommend changes to clinical practice or within health policy decision making - for certain questions, observational studies provide the only evidence - recommendations from observational studies are always stronger when supported by other evidence | |

**S7 Appendix: Modified CASP Qualitative Checklist**

| **#** | **Item** | | **Elaboration** |
| --- | --- | --- | --- |
| Section A: Are the results of the study valid? | | | |
| 1 | Was there a clear statement of the aims of the research?  Yes  Can’t tell  No | HINT: Consider:   - what was the goal of the research - why it was thought important - its relevance | |
| 2 | Is a qualitative methodology appropriate?  Yes  Can’t tell  No | HINT: Consider:   - If the research seeks to interpret or illuminate the actions and/or subjective experiences of research participants - Is qualitative research the right methodology for addressing the research goal | |
| 3 | Was the research design appropriate to address the aims of the research?  Yes  Can’t tell  No | HINT: Consider:   - if the researcher has justified the research design (e.g. have they discussed how they decided which method to use) | |
| 4 | Was the recruitment strategy appropriate to the aims of the research?  Yes  Can’t tell  No | HINT: Consider:   - If the researcher has explained how the participants were selected: - If they explained why the participants they selected were the most appropriate to provide access to the type of knowledge sought by the study - If there are any discussions around recruitment (e.g. why some people chose not to take part) | |
| 5 | Was the data collected in a way that addressed the research issue?  Yes  Can’t tell  No | HINT: Consider:   - If the setting for the data collection was justified - If it is clear how data were collected (e.g. focus group, semi-structured interview etc.) - If the researcher has justified the methods chosen - If the researcher has made the methods explicit (e.g. for interview method, is there an indication of how interviews are conducted, or did they use a topic guide) - If methods were modified during the study. If so, has the researcher explained how and why - If the form of data is clear (e.g. tape recordings, video material, notes etc.) - If the researcher has discussed saturation of data | |
| 6 | Has the relationship between researcher and participants been adequately considered?  Yes  Can’t tell  No | HINT: Consider:   - If the researcher critically examined their own role, potential bias and influence during (a) formulation of the research questions (b) data collection, including sample recruitment and choice of location - How the researcher responded to events during the study and whether they considered the implications of any changes in the research design | |
| Section B: What are the results? | | | |
| 7 | Have ethical issues been taken into consideration?    Yes  Can’t tell  No | HINT: Consider:   - If there are sufficient details of how the research was explained to participants for the reader to assess whether ethical standards were maintained - If the researcher has discussed issues raised by the study (e.g. issues around informed consent or confidentiality or how they have handled the effects of the study on the participants during and after the study) - If approval has been sought from the ethics committee | |
| 8 | Was the data analysis sufficiently rigorous?  Yes  Can’t tell  No | HINT: Consider:   - If there is an in-depth description of the analysis process - If thematic analysis is used. If so, is it clear how the categories/themes were derived from the data - Whether the researcher explains how the data presented were selected from the original sample to demonstrate the analysis process - If sufficient data are presented to support the findings - To what extent contradictory data are taken into account - Whether the researcher critically examined their own role, potential bias and influence during analysis and selection of data for presentation | |
| 9 | Is there a clear statement of findings?  Yes  Can’t tell  No | HINT: Consider whether:   - If the findings are explicit - If there is adequate discussion of the evidence both for and against the researcher’s arguments - If the researcher has discussed the credibility of their findings (e.g. triangulation, respondent validation, more than one analyst) - If the findings are discussed in relation to the original research question | |
| Section C: Will the results help locally? | | | |
| 10 | How valuable is the research?  [free text] | HINT: Consider:   - If the researcher discusses the contribution the study makes to existing knowledge or understanding (e.g. do they consider the findings in relation to current practice or policy, or relevant research based literature - If they identify new areas where research is necessary - If the researchers have discussed whether or how the findings can be transferred to other populations or considered other ways the research may be used | |

**S8 Appendix: Thickness rating**

| **Item** | **Rating** |
| --- | --- |
| *Interpretation in context*  To what extent does the study interpret rather than just describe phenomena? | 1 - not at all effective  2 - slightly effective  3 - effective  4 - very effective |
| *Capturing thoughts and emotions*  To what extent does the study do this, rather than just capturing (for instance) participants' actions? | 1 - not at all effective  2 - slightly effective  3 - effective  4 - very effective |
| *Verisimilitude*  To what extent does the study contain rich detail, making the reader feel as though they are "actually there" in the study setting? Does it make the setting "come alive"? | 1 - not at all effective  2 - slightly effective  3 - effective  4 - very effective |
| *Assigning motivations and intentions*  To what extent does the study explain the reasons, aspirations and goals underlying participants' actions? | 1 - not at all effective  2 - slightly effective  3 - effective  4 - very effective |
| *Explaining meaningfulness of situation*  To what extent does the study draw useful conclusions about the nature of the studied phenomena, comment on whether its findings fit in with or challenge existing evidence and beliefs, or offer new insights into phenomena which have not before been explored in-depth? | 1 - not at all effective  2 - slightly effective  3 - effective  4 - very effective |

**S9 Appendix: Characteristics of included studies**

| **Author (year)** | **Region** | **Country (setting)** | **Theory** | **Quantitative methods** | **Qualitative methods** | **Outcomes** | **Sample characteristics** | **Funding** | **Authorship** |
| --- | --- | --- | --- | --- | --- | --- | --- | --- | --- |
| **Qualitative studies** | | | | | | | | | |
| Gough (2008) | Eastern Africa | Zambia (Lusaka) | Mobilities paradigm |  | Focus groups  In-depth interviews | Mobility and travel behaviour | Sample size: n=60 individuals  Area: urban  Sex: males and females  Age: adolescents and younger adults | Council for Development Research of the Danish International Development Agency | First author: Denmark  Single author study |
| Benwell (2009) | Southern Africa | South Africa (Cape Peninsula) | Mobilities paradigm |  | Focus groups  Art-based methods (drawing, painting, photography, drama)  Mapping  Accompanied walks | Mobility and travel behaviour | Sample size: n=65 individuals  Area: urban  Sex: 40% males, 60% females  Age: [[38](#_ENREF_38),[39](#_ENREF_39),[48-50](#_ENREF_48),[53](#_ENREF_53),[58](#_ENREF_58),[70](#_ENREF_70),[74-76](#_ENREF_74),[95](#_ENREF_95),[100](#_ENREF_100),[113](#_ENREF_113),[114](#_ENREF_114),[132](#_ENREF_132),[133](#_ENREF_133)] | United Kingdom Economic and Social Research Council | First author: United Kingdom  Single author study |
| Kamuhanda (2009) | Eastern Africa | Uganda (Kampala) | None specified |  | Structured interviews | Paratransit | Sample size: n=161 individuals  Area: urban  Sex: males and females  Age: adults | International Labour Organisation  United Kingdom Department for International Development | First author: Uganda  Last author: India |
| Langevang (2009) | Western Africa | Ghana (Madina) | Mobilities paradigm |  | Focus groups  Life histories  Photo-elicitation  Accompanied walks | Mobility and travel behaviour | Sample size: n=17 focus groups  Area: urban  Sex: males and females  Age: adolescents and younger adults | Council for Development Research of the Danish International Development Agency | First author: Denmark  Last author: Denmark |
| Porter (2010) | Eastern Africa  Western Africa  Southern Africa | Ghana (Abura)  Malawi (Ndirande)  South Africa (Ngangalizwe) | Mobilities paradigm |  | In-depth interviews  Focus groups  Life histories  Accompanied walks | Mobility and travel behaviour | Sample size: not reported  Area: urban  Sex: males and females  Age: children and adolescents | United Kingdom Department for International Development  United Kingdom Economic and Social Research Council | First author: United Kingdom  Last author: Ghana |
| Porter (2010) | Southern Africa | South Africa (OR Tambo District Municipality) | None specified |  | In-depth interviews  Focus groups  Life histories  Accompanied walks | Travel behaviour | Sample size: not reported  Area: rural  Sex: males and females  Age: children and adolescents | No funding reported | First author: United Kingdom  Last author: South Africa |
| Lucas (2011) | Southern Africa | South Africa (Tshwane Metropolitan Region) | Social exclusion |  | Focus groups | Travel behaviour | Sample size: n=102 individuals  Area: urban  Sex: males and females  Age: adolescents and adults | South Africa Department of Transport | First author: United Kingdom  Single author study |
| Porter (2011) | Eastern Africa  Western Africa | Ghana  Malawi  Nigeria | Gendered discourse |  | In-depth interviews | Travel behaviour | Sample size: not reported  Area: rural  Sex: 100% female  Age: children and adults | United Kingdom Department for International Development  United Kingdom Economic and Social Research Council | First author: United Kingdom  Single author study |
| Archambault (2012) | Eastern Africa | Mozambique (Inhambane) | None specified |  | Group discussions  Individual conversations | Travel behaviour | Sample size: not reported  Area: urban  Sex: 100% male  Age: younger adults | Social Sciences and Humanities Research Council of Canada  United Kingdom Overseas Research Student Scheme  Central Research Fund, University of London, United Kingdom | First author: United Kingdom  Single author study |
| Turner (2012) | Eastern Africa | Mozambique (Nampula)  Rwanda (Kigali) | None specified |  | Group (household) discussions | Travel behaviour | Sample size: not reported  Area: urban  Sex: not reported  Age: not reported | No funding reported | First author: United Kingdom  Last author: United Kingdom |
| van Blerk (2013) | Southern Africa | South Africa (Cape Town, Cape Flats) | Mobilities paradigm |  | Unstructured interviews  Focus groups  Participatory mapping | Mobility and travel behaviour | Sample size: n=50 individuals  Area: urban  Sex: mostly male  Age: children, adolescents and younger adults | United Kingdom Economic and Social Research Council | First author: United Kingdom  Single author study |
| Raynor (2014) | Eastern Africa | Uganda (Kampala) | None specified |  | Focus groups  In-depth interviews | Paratransit | Sample size: not reported  Area: urban  Sex: not reported  Age: not reported | No funding reported | First author: Uganda  Single author study |
| Alando (2016) | Eastern Africa | Kenya (Kisumu) | None specified |  | Semi-structured interviews  Field notes  Policy analysis | Cycling | Sample size: 2 individuals  Area: urban  Sex: not reported  Age: adults | German Academic Exchange Program  Kenya National Council for Science, Technology and Innovation | First author: Germany  Last author: Germany |
| El-Dorghamy (2016) | Northern Africa | Egypt (Ezbet El-Haggana) | Theory of planned behaviour |  | Focus groups  Semi-structured interviews  Field notes  Photo-elicitation | Travel behaviour | Sample size: n=10 focus groups each comprising 4-11 individuals  Area: urban  Sex: males and females  Age: children and adults | No funding reported | First author: Germany  Last author: United Arab Emirates |
| Esson (2016) | Western Africa | Ghana (Accra) | Mobilities paradigm |  | Focus groups  Semi-structured interviews | Mobility and travel behaviour | Sample size: n=118 individuals, n=14 focus groups  Area: urban  Sex: males and females  Age: adolescents and adults | RurbanAfrica funded by the European Union | First author: United Kingdom  Last author: Ghana |
| Zolnikov (2016) | Eastern Africa | Kenya (Narok) | None specified |  | Accompanied walks | Walking to collect water | Sample size: not reported  Area: rural  Sex: 100% female  Age: adults | No funding reported | First author: United States of America  Single author study |
| Yankson (2017) | Western Africa | Ghana (Sekondi–Takoradi) | Kaufmann's typology of mobility |  | Focus groups  Semi-structured interviews | Travel behaviour | Sample size: n=108 individuals, n=20 focus groups  Area: urban  Sex: males and females  Age: adolescents and adults | RurbanAfrica funded by the European Union | First author: Ghana  Last author: Ghana |
| Gwaka (2018) | Eastern Africa | Zimbabwe (Beitbridge district) | Mobilities paradigm |  | Focus groups  Individual conversations | Mobility and travel behaviour | Sample size: 3 focus groups  Area: rural  Sex: males and females  Age: adolescents and younger adults | Makerere University School of Public Health's Resilient Africa Network  University of Pretoria Southern Africa Resilience Innovation Lab  United States Agency for International Development | First author: South Africa  Single author study |
| Lesteven (2018) | Eastern Africa  Southern Africa | Ethiopia (Addis Ababa)  Kenya (Nairobi) South Africa (Cape Town) | None specified |  | Semi-structured interviews  Field notes | Paratransit and public transport | Sample size: n=32 individuals  Area: urban  Sex: not reported  Age: not reported | Sustainable Mobility Institute Renault-ParisTech, as part of the NexMob research project undertaken by City Mobility Transport Lab | First author: France  Last author: France |
| Poku-Boansi (2018) | Western Africa | Ghana (Kumasi) | None specified |  | Semi-structured interviews | Travel behaviour | Sample size: n=200 individuals  Area: urban  Sex: 48% males, 52% females  Age: adults | No funding reported | First author: Ghana  Last author: Ghana |
| Mixed-method studies | | | | | | | | | |
| Diaz Olvera (2010) | Central Africa  Western Africa | Cameroon (Douala)  Niger (Niamey) | None specified | Cross-sectional survey | In-depth interviews | Use of travel modes | Sample size: not reported  Area: urban  Sex: not reported  Age: not reported | No funding reported | First author: France  Last author: Cameroon |
| Integrated Transport Planning (2010) | Eastern Africa | Uganda (Greater Kampala Metropolitan Area) | None specified | Cross-sectional survey  Traffic counts | Semi-structured interviews | Travel time  Travel distance  Travel speed  Use of travel modes | Sample size: n=126,895 inbound traffic counts; 127,197 outbound traffic counts  Area: urban  Sex: not reported  Age: not reported | No funding reported | First author: United Kingdom  Single author study |
| Nkurunziza (2010) | Eastern Africa | Tanzania (Dar es Salaam) | Behavioural change transactional model | Cross-sectional survey | Interview | Travel distance  Use of travel modes  Travel purpose | Sample size: n=598 individuals  Area: urban  Sex: 75% males, 25% females  Age: children and adults | The Netherlands Cycling Academic Network and Interface for Cycling Expertise | First author: The Netherlands  Last author: The Netherlands |
| Abane (2011) | Western Africa | Ghana (Accra, Kumasi, Tamale and Sekondi–Takoradi) | None specified | Cross-sectional survey | In-depth interviews  Field notes | Paratransit and public transport | Sample size: n=926 individuals, n=21 transport officials  Area: urban  Sex: 51% males, 48% females  Age: adolescents and adults | No funding reported | First author: Ghana  Single author study |
| Amoako-Sakyi (2011) | Western Africa | Ghana (urban, peri-urban and rural communities) | None specified | Cross-sectional survey | Focus groups  In-depth interviews | Cycling | Sample size: n=1005 individuals, n=323 interviews, n=31 focus groups  Area: urban and rural  Sex: 47% males, 53% females  Age: children and adolescents | No funding reported | First author: Ghana  Last author: Ghana |
| Kumar (2011) | Central Africa  Eastern Africa  Western Africa | Cameroon (Douala)  Nigeria (Lagos)  Uganda (Kampala) | Political economy perspective | Cross-sectional survey | Focus groups  Semi-structured interviews  Field notes | Travel distance  Trips  Use of travel modes | Sample size: not reported  Area: urban  Sex: males and females  Age: not reported | European Commission  Islamic Development Bank  African Development Bank  World Bank | First author: not reported  Single author study |
| Porter (2011) | Eastern Africa | Malawi | None specified | Cross-sectional survey | In-depth interviews  Focus groups  Life histories  Accompanied walks | Travel distance  Use of travel modes  Travel purpose | Sample size: n=1,003 individuals  Area: urban and rural  Sex: males and females  Age: children and adolescents | United Kingdom Department for International Development  United Kingdom Economic and Social Research Council | First author: United Kingdom  Last author: Malawi |
| Bogale (2012) | Eastern Africa | Ethiopia (Bahir Dar and Hawassa) | None specified | Cross-sectional survey | In-depth interviews  Field notes | Cycling | Sample size: n=408 individuals, n=32 key informants  Area: urban  Sex: 76% males, 24% females  Age: adolescents and adults | No funding reported | First author: Ethiopia  Single author study |
| Kola (2012) | Eastern Africa | Kenya (Kisumu) | None specified | Cross-sectional survey  Land use maps | Focus groups  Semi-structured interviews  Field notes | Use of travel modes  Travel purpose | Sample size: n=400 households  Area: urban  Sex: not reported  Age: not reported | No funding reported | First author: Kenya  Last author: Kenya |
| Porter (2012) | Eastern Africa  Western Africa  Southern Africa | Ghana  Malawi  South Africa | None specified | Cross-sectional survey | In-depth interviews  Focus groups  Life histories  Accompanied walks | Travel time  Use of travel modes  Travel purpose | Sample size: n~3,000 individuals  Area: urban and rural  Sex: males and females  Age: children and adolescents | United Kingdom Department for International Development  United Kingdom Economic and Social Research Council | First author: United Kingdom  Single author study |
| Diaz Olvera (2013) | Central Africa  Western Africa | Burkina Faso (Ouagadougou)  Cameroon (Douala)  Guinea (Conakry)  Mali (Bamako)  Niger (Niamey)  Senegal (Dakar) | None specified | Cross-sectional survey | Semi-structured interviews | Travel time  Travel distance  Trips  Use of travel modes  Travel purpose | Sample size: n=18,374 individuals, n=60 interviews  Area: urban  Sex: males and females  Age: adolescents and adults | No funding reported | First author: France  Last author: France |
| Porter (2013) | Eastern Africa | Tanzania (Kibaha district) | None specified | Cross-sectional survey | Semi-structured interviews | Travel time  Use of travel modes  Travel purpose | Sample size: n=339 individuals  Area: rural  Sex: 39% males, 61% females  Age: older adults | Africa Community Access Programme | First author: United Kingdom  Last author: Tanzania |
| Ojo (2014) | Western Africa | Ghana (Accra-Takoradi) | None specified | Cross-sectional survey | In-depth interviews  Field notes | Public transport | Sample size: n=497 individuals, n=42 interviews  Area: urban  Sex: 57% males, 43% females  Age: adults | No funding reported | First author: Ghana  Last author: Ghana |
| Agyemang (2015) | Western Africa | Ghana (Greater Accra Metropolitan Area) | None specified | Cross-sectional survey | In-depth interviews | Paratransit and public transport | Sample size: n=30 individuals, n=6 drivers, n=4 key informants, n=120 trips  Area: urban  Sex: individuals 40% males, 60% females  Age: adolescents and adults | No funding reported | First author: Ghana  Single author study |
| Vermeiren (2015) | Eastern Africa | Uganda (Kampala) | Hägerstrand's space-time framework | Cross-sectional survey | Semi-structured interviews  Field notes  Participatory mapping | Travel time  Travel distance  Use of travel modes | Sample size: n=143 individuals  Area: urban  Sex: not reported  Age: adults | Belgium Fund for Scientific Research Flanders | First author: Belgium  Last author: Belgium |
| Mbara (2016) | Southern Africa | South Africa (Johannesburg) | None specified | Cross-sectional survey | Unstructured interviews | Tuk tuk | Sample size: n=150 individuals  Area: urban  Sex: 36% males, 64% females  Age: adults | No funding reported | First author: South Africa  Single author study |
| Seedhouse (2016) | Western Africa | Nigeria (Kwara, Nasarawa, Plateau, Borno, Taraba, Yobe, Kaduna, Zamfara, Abia, Ebonyi, Enugu, Cross River, Edo, Ogun, Ondo and Oyo states) | None specified | Cross-sectional survey | In-depth interviews | Use of travel modes | Sample size: n=92 individuals  Area: rural  Sex: female only  Age: adults | No funding reported | First author: United Kingdom  Last author: United Kingdom |
| Andreasen (2017) | Eastern Africa | Tanzania (Dar es Salaam) | None specified | GIS analysis | Focus groups  Semi-structured interviews  Key informant interviews  Field notes | Travel time  Use of travel modes  Travel purpose | Sample size: n=174 individuals  Area: urban  Sex: males and females  Age: adults | RurbanAfrica funded by the European Union | First author: Denmark  Last author: Denmark |
| Porter (2017) | Southern Africa | South Africa (Eastern Cape and Gauteng Province) | None specified | Cross-sectional survey | In-depth interviews  Focus groups  Life histories  Accompanied walks | Use of travel modes | Sample size: n=642 individuals, n~200 interviews  Area: urban  Sex: males and females  Age: children, adolescents and younger adults | United Kingdom Department for International Development  United Kingdom Economic and Social Research Council | First author: United Kingdom  Last author: South Africa |
| Delatte (2018) | Northern Africa | Algeria (Algiers) Morocco (Casablanca) | Maslow's hierarchical pyramid of human needs | Cross-sectional survey | Focus groups | Public transport | Sample size: n=433 individuals  Area: urban  Sex: males and females (focus groups female only)  Age: adolescents and adults | No funding reported | First author: United Arab Emirates  Last author: United Arab Emirates |
| Evans (2018) | Eastern Africa | Uganda (Kampala) | None specified | Cross-sectional survey  GIS analysis | Semi-structured interviews  Photo elicitation | Paratransit | Sample size: n=60 individuals, n=400 GPS tracks  Area: urban  Sex: males and females  Age: not reported | National Geographic | First author: United Kingdom  Last author: Germany |
| Irlam (2018) | Southern Africa | South Africa (Masiphumelele) | Kaufmann’s ‘motility’ concept | Cross-sectional survey | Focus groups | Cycling | Sample size: n=100 individuals, n=1 focus group  Area: urban  Sex: males and females  Age: adults | No funding reported | First author: South Africa  Single author study |
| Oviedo (2017) | Western Africa | Nigeria (Abuja, Kaduna and Ibadan) | Person-centred framework linking transport and wellbeing | Cross-sectional survey | Semi-structured interviews  In-depth interviews | Use of travel modes  Travel purpose | Sample size: n=337 individuals, n=25 interviews  Area: urban  Sex: 59% males, 41% females  Age: adults | United Kingdom Department for International Development  ICF International  STO Associates, Nigeria | First author: United Kingdom  Last author: United Kingdom |
| Janusz (2019) | Eastern Africa | Uganda (Kampala) | Hägerstrand's model of timespace constraints | GIS analysis | Semi-structured interviews  Key informant interviews  Field notes | Travel time  Travel distance  Trips  Use of travel modes  Travel purpose | Sample size: n=10 individuals  Area: urban  Sex: 30% males, 70% females  Age: adults | No funding reported | First author: Belgium  Last author: Belgium |
| Quantitative studies | | | | | | | | | |
| Aworemi (2008) | Western Africa | Nigeria (Ilorin) |  | Cross-sectional survey |  | Use of travel modes  Travel purpose | Sample size: n=256 individuals  Area: urban  Sex: 66% males, 34% females  Age: adolescents and adults | No funding reported | First author: Nigeria  Last author: Nigeria |
| Bryceson (2008) | Eastern Africa | Ethiopia (Hidabo Abote Wereda and Degem Wereda)  Zambia (Chipata District, Eastern Province and Luanshya District, Copperbelt Province) |  | Cross-sectional survey |  | Travel time  Travel distance  Trips  Use of travel modes | Sample size: not reported  Area: rural  Sex: not reported  Age: not reported | United Kingdom Department for International Development  United Kingdom Transport Research Laboratory | First author: United Kingdom  Last author: United Kingdom |
| Odufuwa (2008) | Western Africa | Nigeria (Lagos) |  | Cross-sectional survey |  | Trips  Use of travel modes | Sample size: n=356 individuals  Area: urban  Sex: males and females  Age: adults | No funding reported | First author: Nigeria  Single author study |
| Bechstein (2010) | Southern Africa | South Africa (Mamelodi and Nellmapius) |  | Cross-sectional survey |  | Trips  Use of travel modes  Travel purpose | Sample size: n=178 individuals  Area: urban  Sex: predominantly male  Age: adults | No funding reported | First author: South Africa  Single author study |
| Behrens (2010) | Southern Africa | South Africa (Cape Town) |  | Cross-sectional survey |  | Use of travel modes | Sample size: n=993 individuals  Area: urban  Sex: 49% males, 51% females  Age: adults | African Centre of Excellence for Studies in Public and Non-motorised Transport funded by Volvo Research and Educational Foundations  Cape Town Metrorail | First author: South Africa  Last author: South Africa |
| Elfiky (2010) | Northern Africa | Egypt (Kafr Elshiekh) |  | Cross-sectional survey |  | Use of travel modes | Sample size: not reported  Area: urban  Sex: males and females  Age: children and adults | No funding reported | First author: Egypt  Single author study |
| Ipingbemi (2010) | Western Africa | Nigeria (Ibadan) |  | Cross-sectional survey |  | Use of travel modes | Sample size: n=264 individuals  Area: urban  Sex: 41% males, 59% females  Age: older adults | No funding reported | First author: Nigeria  Single author study |
| Salon (2010) | Eastern Africa | Kenya (Nairobi) |  | Cross-sectional survey |  | Use of travel modes  Travel purpose | Sample size: n=4,375 individuals  Area: urban  Sex: males and females  Age: children and adults | World Bank  Norwegian Trust Fund | First author: United States of America  Last author: United States of America |
| Alemu (2011) | Western Africa | Sierra Leone |  | Cross-sectional survey |  | Active travel time | Sample size: n=4,997 individuals  Area: not reported  Sex: 46% males, 54% females  Age: adults | No funding reported | First author: Sierra Leone  Last author: Sierra Leone |
| Bwire (2011) | Eastern Africa | Tanzania (Dar Es Salaam) |  | Cross-sectional survey |  | Travel time  Use of travel modes  Travel purpose | Sample size: n=284 individuals  Area: urban  Sex: males and females  Age: children and parents | African Centre of Excellence for Studies in Public and Non-motorised Transport funded by Volvo Research and Educational Foundations | First author: Tanzania  Single author study |
| Babinard (2011) | Northern Africa | Morocco |  | Cross-sectional survey |  | Use of travel modes  Travel purpose | Sample size: n=822 individuals  Area: urban and rural  Sex: 27% males, 73% females  Age: adults | World Bank | First author: United States of America  Single author study |
| Guthold (2011) | Northern Africa  Central Africa  Eastern Africa  Western Africa  Southern Africa | Algeria  Benin  Botswana  Cameroon  Cape Verde  Chad  Cote d'Ivoire  Congo  Eritrea  Ethiopia  Gabon  Madagascar  Malawi  Mali  Mauritania  Mozambique  Niger  Sao Tome et Principe  Sierra Leone  Swaziland  Zambia |  | Cross-sectional survey |  | Active travel time | Sample size: n=57,038 individuals  Area: urban and rural  Sex: 42% males, 58% females  Age: adults | No funding reported | First author: Switzerland  Last author: Switzerland |
| Masaoe (2011) | Eastern Africa  Southern Africa | Kenya (Nairobi)  South Africa (Cape Town)  Tanzania (Dar es Salaam) |  | Cross-sectional survey |  | Trips  Use of travel modes  Travel purpose | Sample size: n=6,013 households  Area: urban  Sex: males and females  Age: children and adults | African Centre of Excellence for Studies in Public and Non-motorised Transport funded by Volvo Research and Educational Foundations | First author: Tanzania  Last author: Kenya |
| Muhammed (2011) | Western Africa | Nigeria (Kano state) |  | Cross-sectional survey |  | Paratransit | Sample size: n=365 individuals  Area: urban  Sex: males and females  Age: adults | No funding reported | First author: Nigeria  Single author study |
| Oyeyemi (2011) | Western Africa | Nigeria (Ibadan) |  | Cross-sectional survey |  | Walking | Sample size: n=1,006 individuals  Area: urban  Sex: 49% males, 51% females  Age: adolescents and adults | No funding reported | First author: Nigeria  Last author: United States of America |
| Venter (2011) | Southern Africa | South Africa (Gauteng) |  | Repeat cross-sectional survey |  | Travel time  Travel distance  Trips  Use of travel modes  Travel purpose | Sample size: n=16,729 individuals  Area: urban  Sex: males and females  Age: adolescents and adults | Gauteng City-Region Observatory funded by University of Johannesburg, University of the Witwatersrand, and the Gauteng Provincial Government | First author: South Africa  Last author: South Africa |
| Venter (2011) | Southern Africa | South Africa |  | Cross-sectional survey |  | Travel time  Use of travel modes  Travel purpose | Sample size: n=163,064 individuals  Area: urban and rural  Sex: males and females  Age: adults | South Africa Department of Transport | First author: South Africa  Single author study |
| Walter (2011) | Southern Africa | South Africa (Nelson Mandela Metropolitan Area) |  | Cross-sectional survey |  | Active travel time | Sample size: n=180 individuals  Area: urban  Sex: 100% females  Age: adults | South Africa National Research Foundation  Nelson Mandela Metropolitan University | First author: South Africa  Last author: South Africa |
| Abiola (2012) | Western Africa | Nigeria (Abeokuta) |  | Cross-sectional survey |  | Trips  Use of travel modes | Sample size: n=1,300 individuals  Area: urban  Sex: not reported  Age: adults | No funding reported | First author: Nigeria  Last author: Nigeria |
| Adetunji (2012) | Western Africa | Nigeria (Ilesa) |  | Cross-sectional survey  Regional and national government datasets |  | Travel time  Travel distance  Trips  Use of travel modes  Travel purpose | Sample size: n=1,200 individuals  Area: urban  Sex: 52% males, 48% females  Age: adults | No funding reported | First author: Nigeria  Single author study |
| Odufuwa (2012) | Western Africa | Nigeria (Ibadan) |  | Cross-sectional survey |  | Travel time  Travel distance  Trips  Use of travel modes  Travel purpose | Sample size: n=203 individuals  Area: urban  Sex: males and females  Age: adults | No funding reported | First author: Nigeria  Last author: Nigeria |
| Salon (2012) | Eastern Africa | Kenya (Nairobi) |  | Cross-sectional survey |  | Travel distance  Use of travel modes | Sample size: n=7,500 individuals  Area: urban  Sex: males and females  Age: children and adults | Volvo Research and Educational Foundation  The Earth Institute at Columbia University, United States of America | First author: United States of America  Last author: Kenya |
| Alade (2013) | Western Africa | Nigeria (Lagos) |  | Cross-sectional survey |  | Travel time  Trips  Use of travel modes  Travel purpose | Sample size: n=155 individuals  Area: urban  Sex: 51% males, 49% females  Age: older adults | No funding reported | First author: Nigeria  Last author: Nigeria |
| Amoh-Gyimah (2013) | Western Africa | Ghana (Kumasi) |  | Cross-sectional survey |  | Travel time  Travel distance  Use of travel modes  Travel purpose | Sample size: n=372 individuals  Area: urban  Sex: 68% males, 32% females  Age: adults | No funding reported | First author: Ghana  Last author: Nigeria |
| Nkurunziza (2013) | Eastern Africa | Tanzania (Dar Es Salaam) |  | Cross-sectional survey |  | Cycling | Sample size: n=448 individuals  Area: urban  Sex: 87% males, 13% females  Age: adolescents and adults | The Netherlands Cycling Academic Network and Interface for Cycling Expertise | First author: The Netherlands  Last author: The Netherlands |
| Dugas (2014) | Eastern Africa  Western Africa  Southern Africa | Ghana (Nkwantakese)  Seychelles  South Africa (Khayelitsha) |  | Cross-sectional survey |  | Active travel time | Sample size: n=1,504 individuals  Area: urban and rural  Sex: males and females  Age: adults | No funding reported | First author: United States of America  Last author: United States of America |
| Gradidge (2014) | Southern Africa | South Africa (Soweto) |  | Cross-sectional survey |  | Active travel time | Sample size: n=977 individuals  Area: urban  Sex: 100% female  Age: adults | Medical Research Council of South Africa  University of the Witwatersrand  United Kingdom Wellcome Trust  United Kingdom Department for International Development  United Kingdom Medical Research Council  The Carnegie Corporation of New York | First author: South Africa  Last author: South Africa |
| Joshi (2014) | Eastern Africa | Kenya (Kibera) |  | Cross-sectional survey |  | Active travel time | Sample size: n=2,061 individuals  Area: urban  Sex: 51% males, 49% females  Age: adults | No funding reported | First author: Kenya  Last author: Kenya |
| Luke (2014) | Eastern Africa  Western Africa  Southern Africa | Ghana  Seychelles  South Africa |  | Cross-sectional survey |  | Active travel time | Sample size: n=1,504 individuals  Area: urban and rural  Sex: males and females  Age: older adults | United States National Institutes of Health | First author: United States of America  Last author: United States of America |
| Mfinanga (2014) | Eastern Africa | Tanzania (Dar es Salaam) |  | Cross-sectional survey |  | Walking | Sample size: n=386 individuals  Area: urban  Sex: 42% males, 58% females  Age: children and adults | No funding reported | First author: Tanzania  Single author study |
| Olawole (2014) | Western Africa | Nigeria (Osogbo) |  | Cross-sectional survey |  | Travel distance  Trips  Use of travel modes  Travel purpose | Sample size: n=250 individuals  Area: urban  Sex: 51% males, 49% females  Age: older adults | No funding reported | First author: Nigeria  Last author: Nigeria |
| Kolbe-Alexander (2015) | Southern Africa | South Africa (Cape Town) |  | Cross-sectional survey |  | Active travel time | Sample size: n=44 individuals  Area: urban  Sex: 23% males, 77% females  Age: older adults | University of Cape Town | First author: South Africa  Last author: South Africa |
| Laverty (2015) | Western Africa  Southern Africa | Ghana  South Africa |  | Cross-sectional survey |  | Active travel time | Sample size: n=9,800 individuals  Area: urban and rural  Sex: males and females  Age: adults | No funding reported | First author: United Kingdom  Last author: United Kingdom |
| Olawole (2015) | Western Africa | Nigeria (Ilesa) |  | Cross-sectional survey |  | Travel time  Travel distance  Trips  Use of travel modes  Travel purpose | Sample size: n=378 individuals  Area: urban  Sex: 62% males, 38% females  Age: older adults | No funding reported | First author: Nigeria  Single author study |
| Salau (2015) | Western Africa | Nigeria (Lagos) |  | Cross-sectional survey |  | Travel time  Travel distance  Use of travel modes | Sample size: n=1,475 individuals  Area: urban  Sex: 60% males, 40% females  Age: adolescents and adults | No funding reported | First author: Nigeria  Single author study |
| Acheampong (2016) | Western Africa | Ghana (Kumasi) |  | Cross-sectional survey |  | Cycling | Sample size: n=550 individuals  Area: urban  Sex: 49% males, 51% females  Age: adults | No funding reported | First author: United Kingdom  Single author study |
| Bartels (2016) | Southern Africa | South Africa (Cape Town) |  | Cross-sectional survey |  | Active travel time  Travel purpose | Sample size: n=1,321 individuals  Area: urban  Sex: males and females  Age: adults | National Research Foundation of South Africa | First author: South Africa  Last author: South Africa |
| Diaz Olvera (2016) | Western Africa | Senegal (Dakar) |  | Repeat cross-sectional survey |  | Travel time  Trips  Use of travel modes  Travel purpose | Sample size: n=22,073 individuals  Area: urban  Sex: males and females  Age: adolescents and adults | Executive Council of Urban Transport in Dakar | First author: France  Last author: France |
| Guwatudde (2016) | Eastern Africa | Uganda |  | Cross-sectional survey |  | Active travel time | Sample size: n=3,987 individuals  Area: urban and rural  Sex: 40% males, 60% females  Age: adults | Uganda government  World Health Organization  World Diabetes Foundation  United Nations Development Program | First author: Uganda  Last author: Uganda |
| Nigatu Haregu (2016) | Eastern Africa | Kenya (Nairobi) |  | Cross-sectional survey |  | Active travel time | Sample size: n=5,190 individuals  Area: urban  Sex: 54% males, 46% females  Age: adults | United Kingdom Wellcome Trust | First author: Kenya  Last author: Kenya |
| Olawole (2016) | Western Africa | Nigeria (Ile-Ife) |  | Cross-sectional survey |  | Travel time  Travel distance  Use of travel modes | Sample size: n=1,638 individuals  Area: urban  Sex: 63% males, 37% females  Age: younger adults | No funding reported | First author: Nigeria  Last author: Nigeria |
| Oyeyemi (2016) | Western Africa | Nigeria (Maiduguri) |  | Cross-sectional survey |  | Active travel time | Sample size: n=1,006 individuals  Area: urban  Sex: 50% males, 50% females  Age: adolescents | No funding reported | First author: Nigeria  Last author: Nigeria |
| Saddier (2016) | Western Africa | Ghana (Greater Accra Metropolitan Area) |  | GIS analysis |  | Paratransit | Sample size: n=580 paratransit routes  Area: urban  Sex: not applicable  Age: not applicable | Canada Fonds Québécois de la Recherche sur la Société et la Culture Nouveaux Chercheurs Program  Canada Research Chairs Program  Canadian Foundation for Innovation | First author: Ghana  Last author: Canada |
| Agyemang (2017) | Western Africa | Ghana (Greater Accra Metropolitan Area) |  | Cross-sectional survey |  | Travel distance  Use of travel modes  Travel purpose | Sample size: n=613 individuals  Area: urban  Sex: males and females  Age: adults | Danish International Development Agency | First author: Ghana  Single author study |
| Groot (2017) | Eastern Africa | Kenya (Nairobi) |  | Cross-sectional survey |  | Active travel time | Sample size: n=10,128 individuals  Area: urban and rural  Sex: males and females  Age: adults | No funding reported | First author: The Netherlands  Last author: Kenya |
| John (2017) | Eastern Africa | Tanzania (Magu district) |  | Cross-sectional survey |  | Active travel time | Sample size: n=5,663 individuals  Area: urban and rural  Sex: 59% males, 41% females  Age: adolescents and adults | No funding reported | First author: Tanzania  Last author: Tanzania |
| Machado-León (2017) | Northern Africa | Algeria (Algiers) |  | Cross-sectional survey |  | Use of travel modes  Travel purpose | Sample size: n=1,454 individuals  Area: urban  Sex: 54% males, 46% females  Age: adolescents and adults | Spanish Initiatives of Development Cooperation Centre, University of Granada | First author: Spain  Last author: Spain |
| Micklesfield (2017) | Southern Africa | South Africa (Agincourt and Soweto) |  | Cross-sectional survey |  | Active travel time | Sample size: n=1,019 individuals  Area: urban and rural  Sex: 100% female  Age: adolescents and adults | Medical Research Council of South Africa  University of the Witwatersrand  South Africa Claude Leon Foundation  United Kingdom Wellcome Trust  United Kingdom Department for International Development  United Kingdom Medical Research Council | First author: South Africa  Last author: South Africa |
| Olawole (2017) | Western Africa | Nigeria (Ile-Ife) |  | Cross-sectional survey |  | Travel distance  Trips  Use of travel modes  Travel purpose | Sample size: n=273 individuals  Area: rural  Sex: 49% males, 51% females  Age: older adults | No funding reported | First author: Nigeria  Single author study |
| Olojede (2017) | Western Africa | Nigeria (Ilesa) |  | Cross-sectional survey |  | Walking | Sample size: n=524 individuals  Area: urban  Sex: 53% males, 47% females  Age: adults | No funding reported | First author: Nigeria  Last author: Nigeria |
| Sabry (2017) | Northern Africa | Egypt (Alexandria) |  | Cross-sectional survey |  | Travel distance  Trips  Use of travel modes  Travel purpose | Sample size: n=182 individuals  Area: urban  Sex: 47% males, 53% females  Age: adults | No funding reported | First author: Egypt  Last author: Egypt |
| Schuyler (2017) | Eastern Africa | Uganda (Rakai) |  | Repeat cross-sectional survey |  | Travel time  Travel purpose | Sample size: n=10,042 observations  Area: urban and rural  Sex: 40% males, 60% females  Age: adolescents and younger adults | United States of America National Institutes of Health | First author: United States of America  Last author: United States of America |
| Acheampong (2018) | Western Africa | Ghana (Tamale) |  | Cross-sectional survey |  | Cycling | Sample size: n=455 individuals  Area: urban  Sex: 59% males, 41% females  Age: adults | No funding reported | First author: Ireland  Last author: Hong Kong |
| Baouni (2018) | Northern Africa | Algeria (Algiers) |  | Cross-sectional survey |  | Public transport | Sample size: n=347 individuals  Area: urban  Sex: 49% males, 51% females  Age: adolescents and adults | No funding reported | First author: Algeria  Last author: Spain |
| Chakwizira (2018) | Southern Africa | South Africa (Gauteng province) |  | Cross-sectional survey |  | Travel time  Use of travel modes  Travel purpose | Sample size: n=1,150 individuals  Area: urban  Sex: males and females  Age: adults | No funding reported | First author: South Africa  Last author: Nigeria |
| El-Sherbiny (2018) | Northern Africa | Egypt (Fayoum governorate) |  | Cross-sectional survey |  | Active travel time | Sample size: n=5,000 individuals  Area: urban and rural  Sex: 46% males, 54% females  Age: adolescents and adults | No funding reported | First author: Egypt  Last author: Egypt |
| Tembe (2018) | Eastern Africa | Kenya (Nairobi)  Mozambique (Maputo) |  | Cross-sectional survey |  | Use of travel modes  Travel purpose | Sample size: n=71,545 individuals  Area: urban  Sex: males and females  Age: adolescents and adults | Japan International Cooperation Agency | First author: Japan  Last author: Japan |
| Davy (2019) | Southern Africa | South Africa (Durban) |  | Cross-sectional survey |  | Travel time | Sample size: n=10 individuals  Area: urban  Sex: 50% males, 50% females  Age: adults | No funding reported | First author: South Africa  Last author: South Africa |
| Oyeyemi (2019) | Western Africa | Nigeria (Maiduguri) |  | Cross-sectional survey |  | Walking | Sample size: n=353 individuals  Area: urban  Sex: 60% males, 40% females  Age: older adults | No funding reported | First author: Nigeria  Last author: Nigeria |

GIS – geographic information system
